# Effectiveness of a New Device for Hand, Wrist, and Forearm Rehabilitation: Feasibility Randomized Controlled Trial

**DOI:** 10.2196/62809

**Published:** 2025-01-27

**Authors:** Adriana M Ríos Rincón, Christine Guptill, Yilina Liubaoerjijin, Mathieu Figeys, Farnaz Koubasi, Geoffrey Gregson, Antonio Miguel Cruz

**Affiliations:** 1 Department of Occupational Therapy, Faculty of Rehabilitation Medicine, University of Alberta, Edmonton, AB, Canada; 2 School of Rehabilitation Sciences, University of Ottawa, Ottawa, ON, Canada; 3 Glenrose Rehabilitation Research, Innovation & Technology Hub, Glenrose Rehabilitation Hospital, Edmonton, AB, Canada; 4 Faculty of Rehabilitation Medicine, University of Alberta, Edmonton, AB, Canada

**Keywords:** hand, upper limb, motion, movement, wrist, therapeutics, rehabilitation, musculoskeletal, musculoskeletal diseases, stroke, wrist fractures, feasibility studies, randomized controlled trial, digital health, physiotherapy, physical therapy, occupational therapy

## Abstract

**Background:**

Forearm, wrist, and hand impairments affect many individuals and impose a significant economic burden on health care systems. The FEPSim (flexion, extension, pronation, and supination) is designed for hand and wrist rehabilitation. It could become part of the standard care for upper extremity rehabilitation, aiming to improve range of motion, dexterity, and strength during therapeutic activities. However, the FEPSim has not yet been tested in a health care setting, highlighting the need for a trial to assess its effectiveness in upper extremity rehabilitation.

**Objective:**

We aim to assess the feasibility of conducting a definitive trial investigating the effectiveness of adding a new device for hand therapy exercises, the FEPSim, to standard care for patients with impairments of the hand, wrist, and forearm.

**Methods:**

Thirty-eight patients with impairments of distal upper extremities were randomly assigned either to the intervention group (FEPSim and standard care, n=19) or to the control group (standard care, n=19). Therapeutic activities to increase strength, range of motion, resistance, and dexterity were delivered by treating hand therapists using the FEPSim device for the intervention group. Outcome measures included wrist passive and active range of motion, grip strength, pinch grip force, and the Patient-Rated Wrist Evaluation.

**Results:**

The trial retention rate (36/38, 95%) and compliance (control group: 100%; intervention group: 89%) were high. The comparisons of the change-from-baseline between groups revealed that in 63.2% (12/19) of the outcome variables, the change was in favor of the FEPSim, with statistically significant improvements in passive wrist flexion (*t*_34_=−0.335, *P*=.008) and grip strength (*t*_34_=−1.841, *P*=.04).

**Conclusions:**

The FEPSim was accepted as part of standard care by therapists and patients at 2 hospitals. The trial design was feasible for hand intervention using the FEPSim device. The FEPSim positively affected grip strength, an objective measure of hand functioning.

## Introduction

Impairments affecting the hand, wrist, and forearm have been found to impact everyday functioning and are associated with reduced quality of life, as well as increased levels of anxiety and depression [1]. These impairments impact a significant proportion of the global population, as they are caused by numerous etiologies such as fractures (including those caused by osteoporosis), osteoarthritis, stroke, and nerve and tendon disorders, among others [1,2].

The existing body of evidence indicates that exercise can lead to varying degrees of improvement in hand, wrist, and forearm impairments, with effects ranging from weak to moderate, particularly in cases of fractures, rheumatic arthritis, and osteoarthritis [1]. Additionally, comprehensive rehabilitation after stroke underscores the crucial role of exercising the hemiparetic hand and wrist across all stages to optimize patient outcomes [3]. However, intervention protocols reported in the literature for exercises as part of hand therapy exhibit significant heterogeneity in conceptualization, dosage, and delivery. This limits the possibility of designing evidence-based clinical guidelines [1,4]. One reason for the observed heterogeneity in hand exercises could be the extensive variety of equipment and materials used during therapy sessions. For example, therapy putty, resistance bands, dumbbells, and jar openers are commonly used. However, these materials typically do not enable therapists to accurately determine and measure their patients’ range of motion or strength during functional hand movements and exercises, which can contribute to inconsistencies in treatment protocols across therapists and settings.

Therapists in clinical settings typically use equipment falling into two main categories to enhance strength, range of motion, and resistance in the hand, wrist, and forearm [5]:

1. Low-cost and portable devices: These devices, averaging US $100, are generally small and designed to specific exercises. Examples are wheel-like or hammer-like devices to exercise pronation and supination and squeezing balls or bars to exercise grip strength. These devices lack direct measurement capabilities during hand therapy activities and are nonadjustable.
2. High-cost electromechanical devices: This category includes both portable and nonportable commercial electromechanical devices whose cost ranges from US $10,000 to US $95,000. Examples are the Simulator II (BTE Technologies) and SaeboReJoyce (Saebo). The BTE Technologies devices have been shown to be a reliable and valid tool, comparable to standard dynamometers, for measuring grip strength in healthy individuals [6,7]. The SaeboReJoyce provides a valid, quantitative, and automated alternative to standardized hand function assessments especially for patients with stroke [8]; however, its effectiveness in improving upper limb function remains inconclusive [9].

There is a clear need for a new rehabilitation device for hand therapy. Existing devices either are low-cost but lack measurement and adaptability or are prohibitively expensive for many health care settings. A versatile, cost-effective device could bridge this gap, offering precision and accessibility for more consistent hand therapy interventions.

The FEPSim (flexion, extension, pronation, and supination [10]), developed by Karma Machining & Manufacturing Ltd, is a rehabilitation device designed explicitly for hand therapy. It targets hand and wrist conditioning through various movements and offers adaptability for different grasp patterns. The device allows therapists to monitor improvements in strength, range of motion, and endurance by enabling controlled resistance adjustments. Resistance is set using a lever on a generic scale from 0 to 1, which corresponds to a torque range of 0.1 to 3.7 newton meters. In addition to resistance, the device can record the repetitions of therapeutic exercises, and is equipped with degree scales that allow therapists to observe patients’ range of motion during active movements of the hand, wrist, or forearm. Thus, although the FEPSim is not an assessment tool, it serves as a useful tool for tracking patient progress in hand therapy. Its potential advantages also include compactness and portability, making it a promising option for rehabilitation of impairments of the hand, wrist, and forearm; however, the effect of using the FEPSim on hand therapy outcomes is unknown. In addition, the device is priced at less than US $3000.

In order to investigate the areas of uncertainty regarding a future definitive randomized controlled trial (RCT), this feasibility study, of which the protocol has been published previously [5], had the following objectives: (1) to assess the methods in terms of recruitment, the eligibility criteria, the type and number of diagnoses included, the length and dosage of the intervention, the data collection methods, and the outcome variables; (2) to explore the clinical effectiveness of adding the FEPSim device to the standard of care for patients with impairments of the hand, wrist, and forearm; and (3) to gather and synthesize the data, from which the sample size of a definitive RCT can be estimated.

## Methods

### Study Design

A pilot or feasibility study is intended to guide the planning of a large-scale investigation. As noted by Thabane et al [11], the primary objective of pilot studies is to evaluate feasibility, mitigating the risk of potentially catastrophic consequences associated with initiating a large-scale study. This precautionary measure aims to prevent the potential “drowning” of the entire research endeavor [11]. A feasibility study serves the purpose of elucidating previously unknown information crucial for designing a definitive RCT. It plays an integral role in acquiring pertinent insights into the acceptance of a novel intervention by both clinicians and patients. Additionally, this study seeks to identify potential beneficiaries of the intervention and elucidate optimal delivery methods within the context of standard care. The decision to undertake a feasibility study is rooted in the purpose of gaining insights into the acceptance and effectiveness of a newly developed hand therapy device which has not yet been tested in health care settings. Additionally, this study aims to evaluate the suitability of the designed research protocol for the clinical settings involved in the investigation.

This study was a feasibility parallel-group RCT that followed the CONSORT (Consolidated Standards of Reporting Trials) statement for randomized feasibility studies. The CONSORT statement serves as a guideline developed to enhance the transparency and quality of reporting in feasibility RCTs [12]. The trial was registered in the ISRCTN (International Standard Randomized Controlled Trial Number; ISRCTN13656014) Registry.

### Ethical Considerations

This study was reviewed and approved by the University of Alberta Health Research Ethics Board - Health Panel (Pro00095587) and the Northern Alberta Clinical Trials and Research Centre. Treating therapists identified eligible participants and invited them to participate. A member of the research team explained the study and obtained signed consent. Participants were able to withdraw from the study at any time, and this did not in any way affect their treatment or their rights as patients at the hospitals. Participants received a coffee shop gift card valued at CAD $25 (US $17.34) upon completing their participation. Personal data were deidentified. The participant names were replaced by numeric codes at the beginning of the study, and these codes were retained throughout data collection and analysis.

### Materials

**Figure 1.**
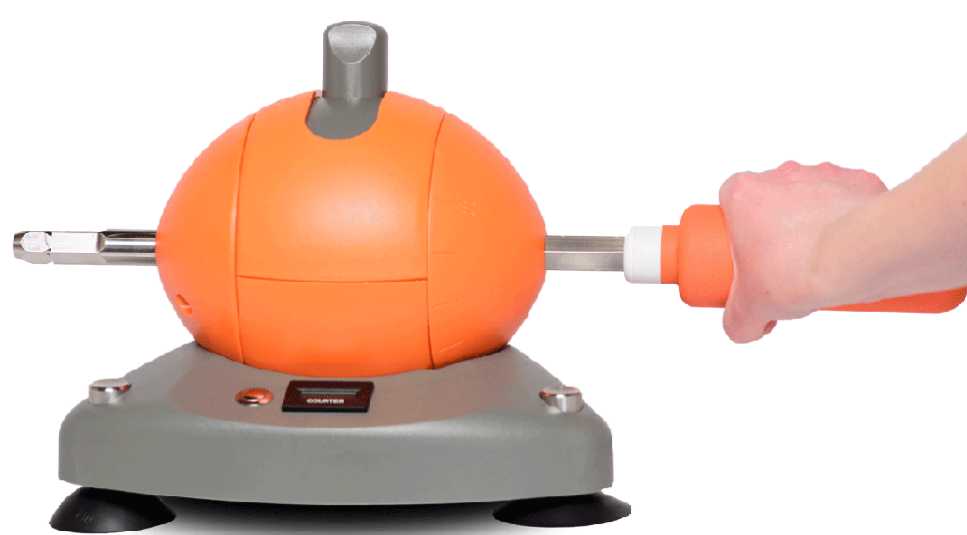
The FEPSim device horizontal model. FEPSim: flexion, extension, pronation, and supination.

The FEPSim is an innovative device designed for the rehabilitation of the hand, wrist, and forearm. This study used the horizontal model depicted in Figure 1. This device features a central structure with an adjustable resistance mechanism, a degree-of-rotation scale for supination and pronation, and a small screen displaying exercise repetition counts. The device is tabletop secure and comprises 2 lateral shafts.

Various attachments facilitate exercises and the simulation of daily activities. For instance, a dowel grip attachment, installable on each shaft, is used for wrist flexion and extension exercises. Rotation and supination exercises involve a 90-degree unit rotation, enabling patients to grasp the dowel and perform clockwise and counter-clockwise rotations. Additional attachments include a key pinch attachment for lateral pinch exercises resembling tasks such as starting a car or turning a key, a T attachment simulating can opener use, a lever attachment for elbow extension or flexion exercises and simulating knife cutting and steering wheel use, and a doorknob attachment for door opening simulations.

### Recruitment

Patients receiving outpatient hand therapy at the Glenrose Rehabilitation Hospital and Royal Alexandra Hospital (Alberta Health Services) who met the inclusion criteria were invited to participate in the trial. The participants were outpatient adults (18 y of age and older) with limitations in their forearm, wrist, or hand function due to distal radial or ulnar fractures, stroke, osteoarthritis (ie, patients who had undergone wrist salvage surgery), Keinbock disease, ulnar shortening, perilunates dislocation, ganglion removal, tumor removals, triangular fibrocartilage complex injury repairs, ligament repair (torn ligament), or spinal cord injury. This study excluded patients with chronic regional pain syndrome, those who experienced unmanaged pain or edema, individuals with communication or cognitive impairments that hindered their ability to understand instructions, and those who were not able to participate in the outpatient hand program at least once a week. The participants were randomly assigned 1:1 to either the experimental group or the control group, using a permuted block-randomization approach with a block size of 4. The stratification was carried out based on diagnosis at each of the hospitals to ensure balance between the groups.

### Procedures

Invitations to participate were posted at several locations in the 2 hospitals. Hand therapists at both hospitals who were unaware of the randomization blocks identified eligible participants. Then, the participants who met inclusion criteria and consented to participate were allocated into one of the groups (experimental group or control group) by the project coordinator who was off-site. This was a single-blinded study. Research assistants blinded to group allocation collected demographics (age, gender, ethnicity, employment status, highest degree or level of education, and medication taken) and outcome measures. Outcome measures were collected at enrollment and weeks 2, 4, 8, and 10.

### Outcome Measures

The primary outcome measures were range of motion and grip strength. Range of motion included active range of motion and passive range of motion of wrist extension, flexion, and radial and ulnar deviation, and pronation and supination were measured using a 12-inch clear plastic goniometer (Baseline 360-degree; Fabrication Enterprises, Inc); grip strength was measured using a hydraulic dynamometer (Baseline Lite hydraulic, 200 lb; Fabrication Enterprises, Inc); pinch strength (including pulp pinch, key grip, or 3 finger grip) was measured using a pinch gauge or pinch meter—60 lb. Grip and pinch strength were measured 3 times, then the mean and SD of the 3 measures were calculated. The secondary outcome variables were the patients’ perceived wrist pain and disability in activities of daily living and was measured with the Patient-Rated Wrist Evaluation (PRWE) questionnaire. The PRWE is a valid and reliable assessment tool of patient-based pain and disability in 3 components: pain; function during specific activities of daily living; and functioning during usual activities (personal care, household work, work, and recreational activities) [5]. Each item is rated from 1 to 10, and the maximum PRWE score is 100. Lower values in the PRWE scale outcome variable are desirable with hand therapy interventions.

### Intervention

#### Overview

Both groups received standard care at each hospital, which consisted of immobilization for 7 to 8 weeks after the time of the injury or surgery (if needed), followed by hand therapy sessions for about 10 weeks. Hand therapy sessions included the management of scar tissue, sensory alterations, and edema, and therapeutic activities to increase strength, range of motion, resistance, and dexterity. For both groups the sessions’ length and frequency depended on the patients’ individualized needs and diagnoses, as determined by their treating therapists. The length of each session was between 30 and 45 minutes, carried out once or twice per week. The sessions were provided by occupational or physical therapists with training and experience in hand therapy.

#### Control Group

The therapeutic activities to increase grip strength and wrist or forearm range of motion were performed using available equipment and materials at each hospital. This included using weights such as dumbbells, elastic and squeezing equipment, therapy putty, bands, and other equipment that imitated the hand patterns required for daily activities (eg, jar openers).

#### Experimental Group

The research team worked with a group of experts to define which equipment and materials used at each hospital for hand rehabilitation could be replaced by the FEPSim device based on its features. As a result, for the experimental group, the therapeutic activities to increase grip strength, and wrist or forearm range of motion were performed using the FEPSim device instead of the equipment and materials used in the control group as described above.

### Statistical Analysis

The analysis was conducted using an intention-to-treat principle. Descriptive statistics were used to characterize the groups at the pre- and posttest. Due to variations in the lengths of the interventions, as a result of various discharge times from treatment services, posttest was defined as the last measure taken once discharge from the outpatient clinic was determined by the hand therapists. Tests of normality were carried out in within and between groups comparisons across the outcome variables as appropriate (ie, 1-tailed paired *t* test or Wilcoxon signed rank test for within group comparisons; independent *t* test or Mann-Whitney *U* test for between group comparisons). We calculated 95% CIs and interpreted the level of uncertainty based on them [13]. The α level of significance was set at *P*<.05 (1-tailed). Cohen *d* values were calculated across variables, and an overall average effect size was calculated using a fixed-effects model for continuous outcomes. All analyses were conducted using SPSS (version 28, IBM Corp). In addition, the minimal clinically important difference (MCID) was calculated. An effect size of 0.2 is commonly considered equivalent to the MCID, so we estimated it by multiplying the SD of the baseline scores by 0.2 (a small effect size) [14].

## Results

### Recruitment

**Figure 2.**
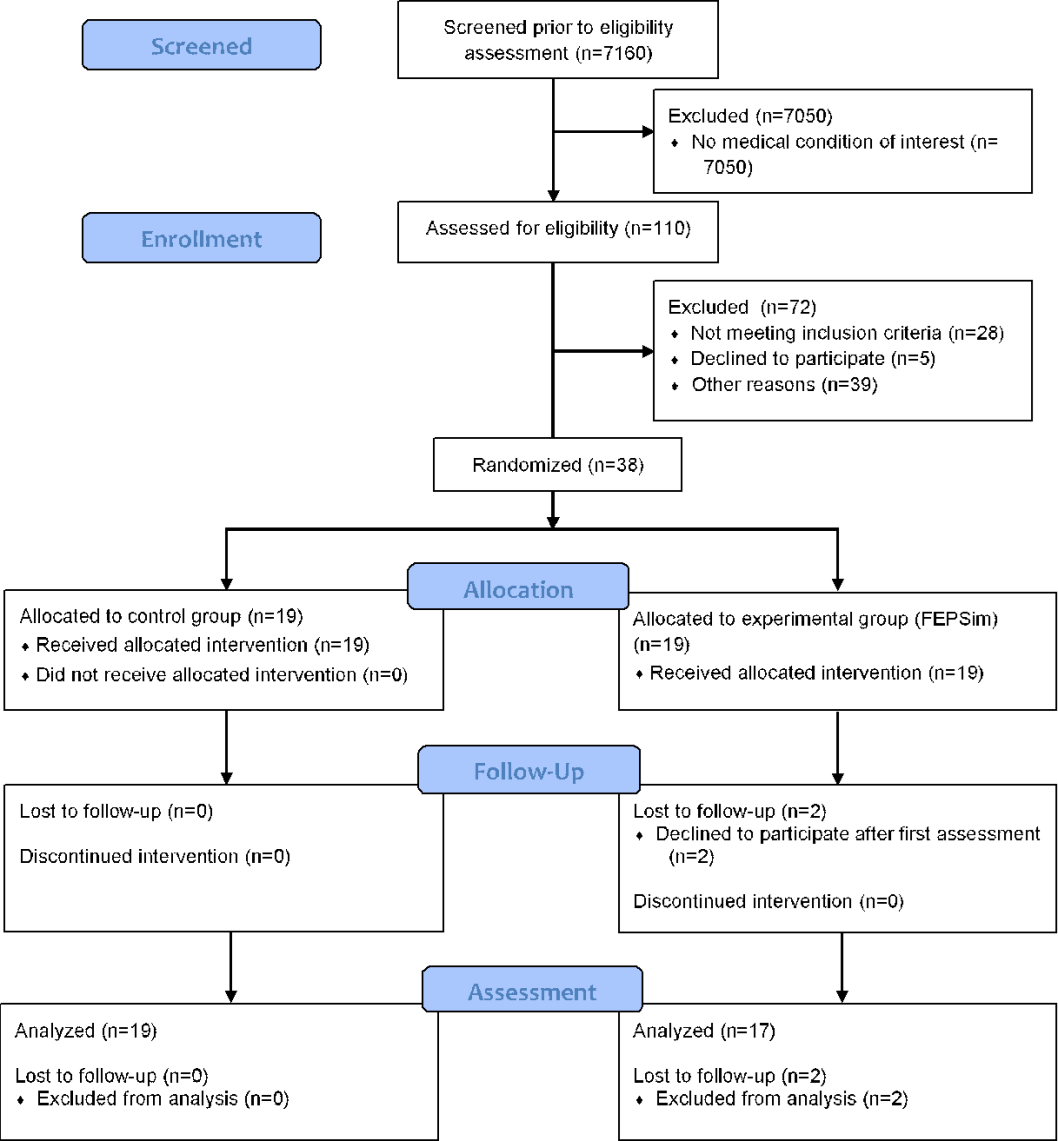
CONSORT (Consolidated Standards of Reporting Trials) flow diagram. FEPSim: flexion, extension, pronation, and supination.

From October 1, 2020, to August 31, 2022, a total of 7160 patients received hand therapy rehabilitation at the 2 hospitals, from which 110 were eligible to enter the trial and 38 consented to participate (participation rate of 35%). The trial retention rate was 95% (36/38). The compliance for the control group was 100%, and for the intervention group, it was 89%. Two participants declined to participate after the first assessment due to disagreement with measures in place to control the spread of the pandemic. Figure 2 presents the flow diagram of the progress during the phases of this study. Table 1 presents the demographics of the 36 participants who completed this study, and Table 2 presents the participants’ baseline characteristics according to group allocation.

**Table 1.** Demographic data per randomized controlled trial group.

Variable		Control group (n=19)	Intervention group (n=17)	Statistics	
				Chi-square (*df*)	*P* value
Age (years), mean (SD)		58.37 (11.09)	54.59 (15.41)	0.851 (34)^a^	.40
**Biological sex, n (%)**				1.3 (1)	.24
	Male	11 (57.9)	13 (76.5)		
	Female	8 (42.1)	4 (23.5)		
**Ethnicity, n (%)**				3.1 (4)	.53
	African-Canadian	1 (5.3)	0 (0)		
	Asian	2 (10.5)	4 (23.5)		
	Caucasian	15 (78.9)	11 (64.7)		
	Latino or Hispanic	0 (0)	1 (5.9)		
	Other or unknown	1 (5.3)	1 (5.9)		
**Highest level of education, n (%)**				6.6 (6)	.36
	Some high school	2 (10.5)	1 (5.9)		
	High school	6 (31.6)	3 (17.6)		
	College or trade school	2 (10.5)	5 (29.4)		
	Bachelor’s degree	7 (36.8)	6 (35.3)		
	Master’s degree	0 (0)	2 (11.8)		
	PhD or higher	1 (5.3)	0 (0)		
	Prefer not to say	1 (5.3)	0 (0)		
**Employment status, n (%)**				3.4 (4)	.48
	Employed	7 (36.8)	7 (41.2)		
	Self-employed	2 (10.5)	0 (0)		
	Unemployed at present	1 (5.3)	0 (0)		
	Retired	6 (31.6)	5 (29.4)		
	Unable to work	3 (15.8)	5 (29.4)		
**Diagnosis, n (%)**				2.0 (4)	.72
	Stroke	9 (47.4)	8 (47.1)		
	Wrist fracture	4 (21.1)	3 (17.6)		
	Osteoarthritis	5 (26.3)	5 (29.4)		
	Ligament repair	1 (5.3)	0 (0)		
	Spinal cord injury	0 (0)	1 (5.9)		

a *t* test (2-tailed).

**Table 2.** Comparison of outcome variables at baseline across groups.

Outcome variables	Control group (n=19)	Intervention group (n=17)	Statistics				
	Mean (SD)	Mean (SD)	*t* test (*df*=34)	*P* value	95% CI	Effect size	Power (%)^a^
Active wrist flexion (degrees)	45.26 (24.98)	42.94 (20.69)	0.302	.38	−13.32 to 17.97	0.101	6
Active wrist extension (degrees)	34.37 (23.47)	33.12 (19.92)	0.171	.43	−13.5 to 16.09	0.057	5.32
Active ulnar deviation (degrees)	21.21 (11.48)	20.24 (11.44)	0.255	.40	−6.80 to 8.75	0.085	5.70
Active radial deviation (degrees)	14.16 (7.91)	16.41 (8.98)	−0.846^b^	.20	−6 to 2	0.266	12.10
Active pronation (degrees)	85.89 (14.18)	86.65 (15.60)	−0.67^b^	.26	−10 to 5	0.051	5.25
Active supination (degrees)	72.16 (27.43)	76.71 (16.40)	−0.127^b^	.45	−15 to 10	0.201	9.01
Passive wrist flexion (degrees)	61.95 (29.94)	55.88 (28.10)	0.624	.27	−13.6 to 25.80	0.209	9.34
Passive wrist extension (degrees)	50.16 (29.98)	51.35 (25.58)	−0.128	.45	−20.1 to 17.80	0.043	5.18
Passive ulnar deviation (degrees)	28.26 (10.96)	25.71 (13.66)	0.623	.27	−5.79 to 10.91	0.206	9.21
Passive radial deviation (degrees)	20.32 (9.29)	23.18 (10.04)	−0.888	.19	−9.41 to 3.68	0.296	13.84
Passive pronation (degrees)	96.68 (17.91)	95.82 (16.65)	0.149	.44	−10.9 to 12.62	0.050	5.24
Passive supination (degrees)	87 (26.10)	89.29 (18.82)	−0.19^b^	.43	−15 to 13	0.101	6
Grip strength (lbs)	33.83 (21.10)	35.32 (25.01)	0^b^	.50	−13.33 to 14.37	0.064	5.40
Three finger grip (lbs)	8.61 (4.25)	9.34 (4.48)	−0.502	.31	−3.69 to 2.23	0.167	7.75
Key grip (lbs)	10.54 (5.07)	11.87 (4.89)	−0.793^b^	.22	−5.20 to 3	0.267	9.05
Pulp pinch (lbs)	7.26 (3.72)	7.97 (3.30)	−0.606	.27	−3.11 to 1.68	0.202	9.05
PRWE^c^ pain sum	17.16 (15.52)	14.47 (11.28)	−0.222^b^	.42	−7 to 12	0.198	8.89
PRWE function sum	57.53 (34.60)	47.47 (29.27)	0.935	.18	−11.79 to 31.90	0.314	14.99
PRWE total	74.68 (47.43)	62 (35.28)	0.901	.19	−15.91 to 41.28	0.303	14.28

a The power (%) is calculated using a statistical equation and reflects the probability of detecting a true effect, not a ratio (eg, n/N). As such, it is presented solely as a percentage and not in the format n (%).

b *z* score.

c PRWE: Patient-Rated Wrist Evaluation.

### Eligibility Criteria

The trial protocol originally proposed to include outpatient adults with 3 specific diagnoses [5]. However, during the intervention sessions, therapists using the FEPSim device identified 8 additional diagnoses that could potentially benefit from its use. As a result, we amended the trial protocol to include patients with a total of 11 diagnoses, as outlined in the Methods section.

### Length and Dosage of the Intervention

Mean numbers of treatment sessions received per group are highlighted in Table 3. We found no statistically significant differences in the number of weeks, the number of sessions, nor the number of days between the pre- and posttest between groups. All the participants in the intervention group received the treatment with the FEPSim. On average, the therapists used the FEPSim in 89% of the sessions in the intervention group.

**Table 3.** Treatment information per group.

Treatment parameters	Control group (n=19)		Intervention group (n=17)		Statistics	
	Minimum, maximum	Mean (SD)	Minimum, maximum	Mean (SD)	*z* score	*P* value
Number of weeks	2, 11.43	7.01 (3.19)	2, 10.86	8.54 (2.75)	−0.779	.21
Number of days (between pre- and posttests)	14, 80	49.05 (22.34)	14, 76	59.76 (19.24)	−0.779	.21
Number of sessions	4, 13	7.94 (2.95)	5, 19	10.82 (4.73)	−1.649	.05
Number of sessions with the FEPSim^a^	0, 0	0 (0)	3, 18	9.76 (4.52)	—^b^	—
Compliance (%)^c^	100, 100	100 (0)	57, 100	89.34 (14.80)	−3.09	<.001

a FEPSim: flexion, extension, pronation, and supination.

b Not applicable.

c Compliance was calculated as the percentage of intervention sessions completed as expected for each participant relative to the total number of prescribed sessions. Since this value varies across participants, the table reports the minimum and maximum values, as well as the mean and SD for each group. Therefore, compliance is presented solely as a percentage and not in the format n (%), as this format is not applicable to the data.

### Data Collection Methods

The overall missing data was very low; from a total of 3204 values across participants and variables, 2.5% of the data was incomplete. Most of the missing values occurred in the baseline data collection phase, which was largely influenced by clinical decisions made by the therapists (ie, if conducting a measurement could compromise the healing process or worsen the clinical condition of a patient). As a result, variables such as pinch strength and grip strength were not measured for 19% (7/36) of participants, while the passive range of motion was not measured for 14% (5/36) of participants at the baseline. In addition, 25% (9/36) of participants did not respond to the “lifting a heavy object” item on the pain subscale of the PRWE at baseline. Most of the missing data were from participants with diagnoses of stroke, wrist fracture, and for ligament repair. Furthermore, the percentage of participants who were discharged from therapy increased as the trial measurement progressed (6%, 25%, and 50% at weeks 4, 8, and 10, respectively); which led the team to define posttest as the last measure taken before discharge.

### Effect of the Intervention

Both groups showed better values in all outcome variables at posttest compared to pretest. The differences between posttest and pretest were statistically significant in 89% (17/19) of the outcome variables for the control group and 95% (18/19) of the outcome variables for the intervention group.

Table 4 displays the change-from-baseline outcome variables for both groups. Comparing the change-from-baseline between groups revealed that in 63% (12/19) of the outcome variables, the change was greater in the intervention group that used the FEPSim than in the control group; however, statistically significant differences between groups were observed in only two of the outcome variables 11% (2/19), specifically grip strength and passive wrist flexion, both in favor of the intervention group. The MCID was 0.96 for grip strength and 1.08 for passive wrist flexion. For the PRWE scale, including the pain and function subscales, as well as the overall summative scale, the change-from-baseline was not in the desired direction (unfavorable for the FEPSim). However, no statistically significant difference was observed for these outcome variables.

**Table 4.** Outcome variables change-from-baseline between the groups.

Outcome variables	Control group (n=19)	Intervention group (n=17)	Statistics				
	Mean (SD)	Mean (SD)	*t *test (*df*=34)	*P* value	95% CI	Effect size	Power (%)^a^
Active wrist flexion (degrees)	10.53 (15.13)	18.53 (14.21)	−1.63	.056	−17.98 to 1.97	0.545	35.46
Active wrist extension (degrees)	9.74 (14.05)	13.71 (12.80)	−0.882	.19	−13.11 to 5.17	0.295	13.81
Active ulnar deviation (degrees)	6.74 (9.92)	5.82 (11.36)	0.257	.40	−6.30 to 8.12	0.086	5.73
Active radial deviation (degrees)	4.26 (6.23)	3.12 (11.11)	−0.397^b^	.35	−5 to 6	0.127	6.57
Active pronation (degrees)	8.37 (12.81)	4.71 (10.83)	−0.682^b^	.25	−6 to 9	0.309	14.63
Active supination (degrees)	12.79 (24.72)	10.29 (13.35)	−0.365^b^	.36	−12 to 11	0.126	6.55
Passive wrist flexion (degrees)	6.74 (17.63)	22.06 (18.70)	−2.530	.008	−27.63 to –3.01	0.843	68.91
Passive wrist extension (degrees)	13.11 (19.08)	14.88 (11.30)	−0.032^b^	.49	−12 to 10	0.113	6.25
Passive ulnar deviation (degrees)	7.63 (9.04)	8.41 (11.72)	−0.365^b^	.36	−8 to 4	0.075	5.54
Passive radial deviation (degrees)	4.84 (7.17)	5.12 (9.70)	−0.098	.46	−6.01 to 5.46	0.033	5.10
Passive pronation (degrees)	9.05 (12.01)	10.06 (10.24)	−0.269	.40	−8.61 to 6.60	0.091	5.80
Passive supination (degrees)	12.00 (18.03)	13.65 (12.56)	−0.314	.38	−12.30 to 9	0.106	6.10
Grip strength (lbs)	7.26 (10.09)	16.06 (17.92)	−1.841	.04	−18.52 to 0.91	0.605	42.14
Three finger grip (lbs)	1.33 (2.67)	2.62 (2.40)	−1.515	.07	−3.02 to 0.44	0.508	31.56
Key grip (lbs)	2.02 (3.16)	2.36 (3.05)	−0.328	.37	−2.45 to 1.77	0.109	6.17
Pulp pinch (lbs)	1.43 (2.32)	2.10 (2.04)	−0.921	.18	−2.16 to 0.81	0.307	14.51
PRWE^c^ pain sum	−2.00 (9.80)	−1.76 (12.20)	−0.064	.48	−7.70 to 7.23	0.022	5.05
PRWE function sum	−29.95 (25.93)	−21.47 (18.78)	−1.111	.14	−23.98 to 7.02	0.375	19.34
PRWE total	−31.95 (31.23)	−23.12 (26.42)	−0.91	.19	−28.55 to 10.89	0.305	14.42

a The power (%) is calculated using a statistical equation and reflects the probability of detecting a true effect, not a ratio (eg, n/N). As such, it is presented solely as a percentage and not in the format n (%).

b *z* score.

c PRWE: Patient-Rated Wrist Evaluation.

### Sample Size of a Future Definitive RCT

The overall fixed-effects model calculated a small effect size. That is, we obtained a statistically significant test for Cohen *d*=0.189 (*z* score=2.450, 95% CI 0.038 to 0.340, *P*=.01) with an SE of 0.077. With a statistical power of 0.8, an α of .05, and the effect size of 0.189, the minimum sample size required for a future definitive RCT is 350 participants (174 participants in each group).

When estimating the effect size per group using a fixed-effects model for continuous outcomes, the overall average effect size for the intervention group had a higher value. That is, we obtained a statistically significant test for Cohen *d*=0.420 (*z* score=5.360, 95% CI 0.267 to 0.574, *P*<.001) with an SE of 0.070 and a *d*=0.310 (*z* score=4.092, 95% CI 0.161 to 0.458, *P*<.001) with an SE of 0.075 for the intervention and control groups, respectively.

## Discussion

### Trial Feasibility

Overall, the research design is feasible for a definitive RCT and offers valuable information for ongoing and future studies. The high trial retention and compliance showed that both the therapists and participants from the 2 hand therapy services agreed to incorporate the FEPSim into their hand therapy treatments. The therapists’ acceptance of this device is also backed up by the qualitative results of another study published elsewhere [15].

### Clinical Effectiveness of Adding the FEPSim Device to Hand Therapy

This study provides evidence of the potential clinical benefits of incorporating the FEPSim device into hand therapy. Both groups improved with the hand therapy treatment in around 8 weeks. However, the FEPSim group showed improvement in more outcome variables, associated with a statistically and significantly higher effect size. For grip strength and passive wrist flexion, the change-from-baseline was statistically significant in favor of the FEPSim. In the intervention group, 76.4% of participants showed a change equal to or greater than the MCID for grip strength, and 88.2% for passive wrist flexion. Grip strength is a commonly used objective proxy of hand function due to its strong correlation with overall hand performance and functionality [16,17]. Grip strength is a composite measure that reflects the integrated function of various hand muscles and joints, capturing both intrinsic and extrinsic muscle function, is clinically relevant and is associated with various health outcomes [18]. Furthermore, sufficient strength in the upper limb is related to the ability to adequately perform many activities of daily living.

The significant improvements in grip strength are clinically relevant for the most common diagnoses included in our trial. Postimmobilization following a wrist fracture often results in stiffness and muscle wasting, leading to a decrease in grip strength, which in turn affects the individuals’ capacity to use the affected hand in daily activities [12]. High movement dosage after distal radius fractures has been found to be critical to increasing grip strength and range of motion. For example, repetitive wrist extension exercises have been found to facilitate regaining grip strength [19]. Additionally, wrist surgical salvage procedures aimed at alleviating osteoarthritis pain have been observed to decrease wrist range of motion and grip strength, potentially leading to long-term impairment [20]. The postoperative rehabilitation goal focuses on pain-free functional wrist motion and includes isometric strengthening exercises, followed by isotonic strengthening and progressive resistive exercises. Depending on the specific surgical salvage procedure, patients can achieve between 53% and 83% of normal contralateral grip strength [21]. Finally, following a stroke, there is a substantial reduction in maximal voluntary force within arm and hand muscles, contributing to significant upper-limb weakness during acute and chronic stages of recovery. A meta-analysis proves that graded strengthening programs improve grip strength and hand function in stroke survivors [22].

The significant improvements in grip strength can be explained by physiology. The use of the FEPSim as part of the therapeutic sessions allowed patients to repeat specific movements such as wrist FEPSim, as well as different grasp patterns with a resistance appropriate to the patient’s progress at each session. This promoted muscle activation and efficiency of muscle recruitment in the wrist and hand intrinsic and extrinsic muscles, leading to an improvement in grip strength. Finally, the diverse wrist and hand movements the device facilitated might improve tendon gliding and elongation, reducing adhesions or tightness and enhancing passive wrist flexion.

The findings underscore the potential efficacy of FEPSim in augmenting grip strength among individuals diagnosed with specific conditions. The device’s multifaceted capabilities, including the provision of real-time repetition counts, adjustable resistance levels, and the simulation of daily tasks impacted by hand injuries (eg, knife cutting), demonstrate its ability to enable multiple repetitions, carry out graded strengthening programs, and perform hand grasp and grip patterns used in functional activities in a controlled and safe manner. These features appear instrumental in optimizing movement dosage, thereby potentially enhancing clinical outcomes in therapeutic interventions.

### Recommendations for a Definitive RCT

Based on the results of this feasibility trial, the authors make the following recommendations for a definitive RCT. First, to investigate a larger sample of diagnoses, including the emerging 8 diagnoses, from the outset to promote sample size and a more robust evaluation. Second, we recommend having 3 measurement points pretest (wk 0), posttest (immediately after discharge), and follow-up at 6 months. This recommendation is based on the observation that the percentage of participants who were discharged from hand therapy services increased during the trial, and that participants may have been discharged at different points during the proposed protocol. Third, considering that the sample size calculation showed that a total of 350 participants will be required and that we had a dropout rate of 10%, a total of 385 participants will be required to participate in a definitive RCT. Considering that the participation rate of this feasibility trial was 34.5%, for a definitive trial the required accessible population will be 1116 patients. To ensure an adequate sample size for a definitive RCT that recruits participants over a 2-year period, it is recommended that recruitment is from 10 settings. This is based on the observation that the feasibility trial admitted approximately 5 patients per month who potentially met the inclusion criteria, resulting in an estimated 47 eligible patients per month (1116 patients/24 mo). It is important to note that these conclusions were reached under challenging circumstances, as the data used to inform this recommendation was collected in hospital settings during the COVID-19 pandemic.

### Study Limitations

It is worth noting that this study has some limitations. Given the limited sample size and low statistical power, caution should be exercised when interpreting the results; it is important to consider these limitations in the context of its feasibility nature and the power calculation conducted for a future definitive trial. Another important consideration is that the 2 variables showing statistically significant differences between groups—grip strength and passive wrist flexion—had missing values in 19% and 14% of participants, respectively. These missing pretest values were imputed using the single imputation method of carrying the last observation backward. While this approach is widely used, it, as with all imputation methods, has inherent limitations. The COVID-19 pandemic had a significant impact on the trial, with different hospital policies for COVID-19 prevention and control, resulting in varying recruitment launch dates and the closure of one hospital’s hand therapy service. This led to a significant reduction in the number of potential participants who met the inclusion criteria. In addition, the data collection was affected, as the trial was conducted over several months during the pandemic. A study by Ivy et al [23] investigated the pandemic’s impact on hand therapy services; they reported a decrease in caseload for 98% of the 719 surveyed hand therapists, with 46% providing telehealth services. However, it is important to note that the impact of the pandemic on the results of this study may differ in a postpandemic world, where hand therapy services may have returned to prepandemic levels. Lastly, as no follow-up measurement points were incorporated in this study, the long-term effects cannot be determined; thus, the results are limited to their generalizability to the short-term.

### Conclusions

To conclude, our study’s findings suggest that it is feasible and acceptable to conduct a future definitive RCT to assess the effectiveness of the FEPSim device in improving impairments of the hand, wrist, and forearm. This trial had high retention and compliance, indicating that the therapists and participants from 2 hand therapy services agreed to incorporate the FEPSim in their hand therapy treatment. The FEPSim had a positive effect on grip strength, an objective measure of hand functioning. The device features appear instrumental in optimizing movement dosage, thereby potentially enhancing clinical outcomes in therapeutic interventions.

## Supplementary material

10.2196/62809 Checklist 1 CONSORT-EHEALTH checklist (V 1.6.1).

## Abbreviations

CONSORT: Consolidated Standards of Reporting Trials
FEPSim: flexion, extension, pronation, and supination
ISRCTN: International Standard Randomized Controlled Trial Number
MCID: minimal clinically important difference
PRWE: Patient-Rated Wrist Evaluation
RCT: randomized controlled trial
