# Supplementary material for: Effectiveness of a New Device for Hand, Wrist, and Forearm Rehabilitation: Feasibility Randomized Controlled Trial
Source: JMIR Rehabil Assist Technol. 2025 Jan 27;12:e62809. doi: 10.2196/62809 (PMC11789689; doi:10.2196/62809)
Supplement: Checklist 1 [file rehab-v12-e62809-s001.pdf]

CONSORT-EHEALTH (V 1.6.1) - Submission/Publication Form

The CONSORT-EHEALTH checklist is intended for authors of randomized trials evaluating web-based and Internet-based applications/interventions, including mobile interventions, electronic games (incl multiplayer games), social media, certain telehealth applications, and other interactive and/or networked electronic applications. Some of the items (e.g. all subitems under item 5 - description of the intervention) may also be applicable for other study designs.

The goal of the CONSORT EHEALTH checklist and guideline is to be
a) a guide for reporting for authors of RCTs,
b) to form a basis for appraisal of an ehealth trial (in terms of validity)

CONSORT-EHEALTH items/subitems are MANDATORY reporting items for studies published in the Journal of Medical Internet Research and other journals / scientific societies endorsing the checklist.

Items numbered 1., 2., 3., 4a., 4b etc are original CONSORT or CONSORT-NPT (non-pharmacologic treatment) items.
Items with Roman numerals (i., ii, iii, iv etc.) are CONSORT-EHEALTH extensions/clarifications.

As the CONSORT-EHEALTH checklist is still considered in a formative stage, we would ask that you also RATE ON A SCALE OF 1-5 how important/useful you feel each item is FOR THE PURPOSE OF THE CHECKLIST and reporting guideline (optional).

Mandatory reporting items are marked with a red *.
In the textboxes, either copy & paste the relevant sections from your manuscript into this form - please include any quotes from your manuscript in QUOTATION MARKS,
or answer directly by providing additional information not in the manuscript, or elaborating on why the item was not relevant for this study.

YOUR ANSWERS WILL BE PUBLISHED AS A SUPPLEMENTARY FILE TO YOUR PUBLICATION IN JMIR AND ARE CONSIDERED PART OF YOUR PUBLICATION (IF ACCEPTED).
Please fill in these questions diligently. Information will not be copyedited, so please use proper spelling and grammar, use correct capitalization, and avoid abbreviations.

DO NOT FORGET TO SAVE AS PDF _AND_ CLICK THE SUBMIT BUTTON SO YOUR ANSWERS ARE IN OUR DATABASE !!!

Citation Suggestion (if you append the pdf as Appendix we suggest to cite this paper in the caption):
Eysenbach G, CONSORT-EHEALTH Group

CONSORT-EHEALTH: Improving and Standardizing Evaluation Reports of Web-based and Mobile Health Interventions
J Med Internet Res 2011;13(4):e126
URL: http://www.jmir.org/2011/4/e126/
doi: 10.2196/jmir.1923
PMID: 22209829

aros@ualberta.ca Switch account

Not shared

Draft saved

* Indicates required question

Your name *

First Last

Adriana María Ríos Rincón

Primary Affiliation (short), City, Country *

University of Toronto, Toronto, Canada

University of Alberta

Your e-mail address *

abc@gmail.com

aros@ualberta.ca

Title of your manuscript *

Provide the (draft) title of your manuscript.

Effectiveness of a new device for hand, wrist, and forearm rehabilitation: A Feasibility Randomized Controlled Trial

Name of your App/Software/Intervention *

If there is a short and a long/alternate name, write the short name first and add the long name in brackets.

FEPSim

Evaluated Version (if any)

e.g. "V1", "Release 2017-03-01", "Version 2.0.27913"

rev20241114_JMIR

Language(s) *

What language is the intervention/app in? If multiple languages are available, separate by comma (e.g. "English, French")

English

URL of your Intervention Website or App

e.g. a direct link to the mobile app on app in appstore (itunes, Google Play), or URL of the website. If the intervention is a DVD or hardware, you can also link to an Amazon page.

https://fepsim.com/

URL of an image/screenshot (optional)

https://fepsim.com/

Accessibility *

Can an enduser access the intervention presently?

- ( ) access is free and open
- ( ) access only for special usergroups, not open
- (•) access is open to everyone, but requires payment/subscription/in-app purchases
- ( ) app/intervention no longer accessible
- ( ) Other:

Primary Medical Indication/Disease/Condition *

e.g. "Stress", "Diabetes", or define the target group in brackets after the condition, e.g. "Autism (Parents of children with)", "Alzheimers (Informal Caregivers of)"

People with limitations in their forearm, wrist, [illegible]

Primary Outcomes measured in trial *

comma-separated list of primary outcomes reported in the trial

Range of motion and grip strength

Secondary/other outcomes

Are there any other outcomes the intervention is expected to affect?

Patients' perceived wrist pain and disability in activities of daily living

Recommended "Dose" *

What do the instructions for users say on how often the app should be used?

- ( ) Approximately Daily
- (•) Approximately Weekly
- ( ) Approximately Monthly
- ( ) Approximately Yearly
- ( ) "as needed"
- ( ) Other:

Approx. Percentage of Users (starters) still using the app as recommended after 3 months *

- (•) unknown / not evaluated
- ( ) 0-10%
- ( ) 11-20%
- ( ) 21-30%
- ( ) 31-40%
- ( ) 41-50%
- ( ) 51-60%
- ( ) 61-70%
- ( ) 71%-80%
- ( ) 81-90%
- ( ) 91-100%
- ( ) Other:

Overall, was the app/intervention effective? *

- ( ) yes: all primary outcomes were significantly better in intervention group vs control
- (x) partly: SOME primary outcomes were significantly better in intervention group vs control
- ( ) no statistically significant difference between control and intervention
- ( ) potentially harmful: control was significantly better than intervention in one or more outcomes
- ( ) inconclusive: more research is needed
- ( ) Other:

Article Preparation Status/Stage *

At which stage in your article preparation are you currently (at the time you fill in this form)

- ( ) not submitted yet - in early draft status
- ( ) not submitted yet - in late draft status, just before submission
- ( ) submitted to a journal but not reviewed yet
- ( ) submitted to a journal and after receiving initial reviewer comments
- (x) submitted to a journal and accepted, but not published yet
- ( ) published
- ( ) Other:

Journal *

If you already know where you will submit this paper (or if it is already submitted), please provide the journal name (if it is not JMIR, provide the journal name under "other")

- ○ not submitted yet / unclear where I will submit this
- ○ Journal of Medical Internet Research (JMIR)
- ○ JMIR mHealth and UHealth
- ○ JMIR Serious Games
- ○ JMIR Mental Health
- ○ JMIR Public Health
- ○ JMIR Formative Research
- ○ Other JMIR sister journal
- ◉ Other: JMIR Rehabilitation and Assistive Technologies

Is this a full powered effectiveness trial or a pilot/feasibility trial? *

- ◉ Pilot/feasibility
- ○ Fully powered

Manuscript tracking number *

If this is a JMIR submission, please provide the manuscript tracking number under "other" (The ms tracking number can be found in the submission acknowledgement email, or when you login as author in JMIR. If the paper is already published in JMIR, then the ms tracking number is the four-digit number at the end of the DOI, to be found at the bottom of each published article in JMIR)

- ○ no ms number (yet) / not (yet) submitted to / published in JMIR
- ◉ Other: 62809

TITLE AND ABSTRACT

1a) TITLE: Identification as a randomized trial in the title

1a) Does your paper address CONSORT item 1a? *

I.e does the title contain the phrase "Randomized Controlled Trial"? (if not, explain the reason under "other")

- (selected) yes
- ( ) Other:

1a-i) Identify the mode of delivery in the title

Identify the mode of delivery. Preferably use “web-based” and/or “mobile” and/or “electronic game” in the title. Avoid ambiguous terms like “online”, “virtual”, “interactive”. Use “Internet-based” only if Intervention includes non-web-based Internet components (e.g. email), use “computer-based” or “electronic” only if offline products are used. Use “virtual” only in the context of “virtual reality” (3-D worlds). Use “online” only in the context of “online support groups”. Complement or substitute product names with broader terms for the class of products (such as “mobile” or “smart phone” instead of “iphone”), especially if the application runs on different platforms.

	1	2	3	4	5	
subitem not at all important	(selected)	( )	( )	( )	( )	essential

Clear selection

Does your paper address subitem 1a-i? *

Copy and paste relevant sections from manuscript title (include quotes in quotation marks "like this" to indicate direct quotes from your manuscript), or elaborate on this item by providing additional information not in the ms, or briefly explain why the item is not applicable/relevant for your study

Not applicable as this is not a mobile APP intervention

1a-ii) Non-web-based components or important co-interventions in title

Mention non-web-based components or important co-interventions in title, if any (e.g., “with telephone support”).

	1	2	3	4	5	
subitem not at all important	◉	○	○	○	○	essential

Clear selection

Does your paper address subitem 1a-ii?

Copy and paste relevant sections from manuscript title (include quotes in quotation marks "like this" to indicate direct quotes from your manuscript), or elaborate on this item by providing additional information not in the ms, or briefly explain why the item is not applicable/relevant for your study

Not applicable as this is not a mobile APP intervention

1a-iii) Primary condition or target group in the title

Mention primary condition or target group in the title, if any (e.g., “for children with Type I Diabetes”) Example: A Web-based and Mobile Intervention with Telephone Support for Children with Type I Diabetes: Randomized Controlled Trial

	1	2	3	4	5	
subitem not at all important	○	○	○	○	◉	essential

Clear selection

Does your paper address subitem 1a-iii? *

Copy and paste relevant sections from manuscript title (include quotes in quotation marks "like this" to indicate direct quotes from your manuscript), or elaborate on this item by providing additional information not in the ms, or briefly explain why the item is not applicable/relevant for your study

"Effectiveness of a new device for hand, wrist, and forearm rehabilitation: A Feasibility Randomized Controlled Trial"

1b) ABSTRACT: Structured summary of trial design, methods, results, and conclusions

NPT extension: Description of experimental treatment, comparator, care providers, centers, and blinding status.

1b-i) Key features/functionalities/components of the intervention and comparator in the METHODS section of the ABSTRACT

Mention key features/functionalities/components of the intervention and comparator in the abstract. If possible, also mention theories and principles used for designing the site. Keep in mind the needs of systematic reviewers and indexers by including important synonyms. (Note: Only report in the abstract what the main paper is reporting. If this information is missing from the main body of text, consider adding it)

	1	2	3	4	5	
subitem not at all important	○	○	○	○	◉	essential

Clear selection

Does your paper address subitem 1b-i? *

Copy and paste relevant sections from the manuscript abstract (include quotes in quotation marks "like this" to indicate direct quotes from your manuscript), or elaborate on this item by providing additional information not in the ms, or briefly explain why the item is not applicable/relevant for your study

"Thirty-eight patients with impairments of distal upper extremity were randomly assigned to either the intervention group (FEPSim® and standard care, n = 19) or to the control group (standard care, n = 19). Therapeutic activities to increase strength, range of motion, resistance, and dexterity were delivered by treating hand therapists using the FEPSim® device for the intervention group."

1b-ii) Level of human involvement in the METHODS section of the ABSTRACT

Clarify the level of human involvement in the abstract, e.g., use phrases like “fully automated” vs. “therapist/nurse/care provider/physician-assisted” (mention number and expertise of providers involved, if any). (Note: Only report in the abstract what the main paper is reporting. If this information is missing from the main body of text, consider adding it)

	1	2	3	4	5	
subitem not at all important	○	○	○	○	◉	essential

Clear selection

Does your paper address subitem 1b-ii?

Copy and paste relevant sections from the manuscript abstract (include quotes in quotation marks "like this" to indicate direct quotes from your manuscript), or elaborate on this item by providing additional information not in the ms, or briefly explain why the item is not applicable/relevant for your study

"Therapeutic activities to increase strength, range of motion, resistance, and dexterity were delivered by treating hand therapists using the FEPSim device for the intervention group."

1b-iii) Open vs. closed, web-based (self-assessment) vs. face-to-face assessments in the METHODS section of the ABSTRACT

Mention how participants were recruited (online vs. offline), e.g., from an open access website or from a clinic or a closed online user group (closed usergroup trial), and clarify if this was a purely web-based trial, or there were face-to-face components (as part of the intervention or for assessment). Clearly say if outcomes were self-assessed through questionnaires (as common in web-based trials). Note: In traditional offline trials, an open trial (open-label trial) is a type of clinical trial in which both the researchers and participants know which treatment is being administered. To avoid confusion, use “blinded” or “unblinded” to indicated the level of blinding instead of “open”, as “open” in web-based trials usually refers to “open access” (i.e. participants can self-enrol). (Note: Only report in the abstract what the main paper is reporting. If this information is missing from the main body of text, consider adding it)

	1	2	3	4	5	
subitem not at all important	(●)	( )	( )	( )	( )	essential

Clear selection

Does your paper address subitem 1b-iii?

Copy and paste relevant sections from the manuscript abstract (include quotes in quotation marks "like this" to indicate direct quotes from your manuscript), or elaborate on this item by providing additional information not in the ms, or briefly explain why the item is not applicable/relevant for your study

Not applicable as this is not a mobile APP intervention

1b-iv) RESULTS section in abstract must contain use data

Report number of participants enrolled/assessed in each group, the use/uptake of the intervention (e.g., attrition/adherence metrics, use over time, number of logins etc.), in addition to primary/secondary outcomes. (Note: Only report in the abstract what the main paper is reporting. If this information is missing from the main body of text, consider adding it)

	1	2	3	4	5	
subitem not at all important	○	○	○	○	◉	essential

Clear selection

Does your paper address subitem 1b-iv?

Copy and paste relevant sections from the manuscript abstract (include quotes in quotation marks "like this" to indicate direct quotes from your manuscript), or elaborate on this item by providing additional information not in the ms, or briefly explain why the item is not applicable/relevant for your study

"The trial retention rate and compliance were high (94.73% and 94.96%, respectively)."

1b-v) CONCLUSIONS/DISCUSSION in abstract for negative trials

Conclusions/Discussions in abstract for negative trials: Discuss the primary outcome - if the trial is negative (primary outcome not changed), and the intervention was not used, discuss whether negative results are attributable to lack of uptake and discuss reasons. (Note: Only report in the abstract what the main paper is reporting. If this information is missing from the main body of text, consider adding it)

	1	2	3	4	5	
subitem not at all important	○	○	○	○	◉	essential

Clear selection

Does your paper address subitem 1b-v?

Copy and paste relevant sections from the manuscript abstract (include quotes in quotation marks "like this" to indicate direct quotes from your manuscript), or elaborate on this item by providing additional information not in the ms, or briefly explain why the item is not applicable/relevant for your study

"The comparisons between groups of the change-from-baseline revealed that in 63.2% (12/19) of the outcome variables, the change was in favor of the FEPSim, with statistically significant improvements in passive wrist flexion (t(34)=-0.335, p=.008) and grip strength (t(34)=-1.841, p=.037)."

INTRODUCTION

2a) In INTRODUCTION: Scientific background and explanation of rationale

2a-i) Problem and the type of system/solution

Describe the problem and the type of system/solution that is object of the study: intended as stand-alone intervention vs. incorporated in broader health care program? Intended for a particular patient population? Goals of the intervention, e.g., being more cost-effective to other interventions, replace or complement other solutions? (Note: Details about the intervention are provided in “Methods” under 5)

	1	2	3	4	5	
subitem not at all important	○	○	○	○	●	essential

Clear selection

Does your paper address subitem 2a-i? *

Copy and paste relevant sections from the manuscript (include quotes in quotation marks "like this" to indicate direct quotes from your manuscript), or elaborate on this item by providing additional information not in the ms, or briefly explain why the item is not applicable/relevant for your study

"Existing devices are either low-cost but lack measurement and adaptability or are prohibitively expensive for many healthcare settings. A versatile, cost-effective device could bridge this gap, offering precision and accessibility for more consistent hand therapy interventions."

2a-ii) Scientific background, rationale: What is known about the (type of) system

Scientific background, rationale: What is known about the (type of) system that is the object of the study (be sure to discuss the use of similar systems for other conditions/diagnoses, if appropiate), motivation for the study, i.e. what are the reasons for and what is the context for this specific study, from which stakeholder viewpoint is the study performed, potential impact of findings [2]. Briefly justify the choice of the comparator.

	1	2	3	4	5	
subitem not at all important	○	○	○	○	◉	essential

Clear selection

Does your paper address subitem 2a-ii? *

Copy and paste relevant sections from the manuscript (include quotes in quotation marks "like this" to indicate direct quotes from your manuscript), or elaborate on this item by providing additional information not in the ms, or briefly explain why the item is not applicable/relevant for your study

"Although the FEPSim is not an assessment tool, it serves as a useful tool for tracking patient progress in hand therapy. Its potential advantages also include compactness and portability, making it a promising option for rehabilitation of impairments of the hand, wrist, and forearm; however, the effect of using the FEPSim on hand therapy outcomes is unknown."

2b) In INTRODUCTION: Specific objectives or hypotheses

Does your paper address CONSORT subitem 2b? *

Copy and paste relevant sections from the manuscript (include quotes in quotation marks "like this" to indicate direct quotes from your manuscript), or elaborate on this item by providing additional information not in the ms, or briefly explain why the item is not applicable/relevant for your study

"Objectives: (1) to assess the methods in terms of recruitment, the eligibility criteria, the type and number of diagnoses included, the length and dosage of the intervention, the data collection methods, and the outcome variables; (2) to explore the clinical effectiveness of adding the FEPSim device to the standard of care for patients with impairments of the of the hand, wrist, and forearm; (3) to gather and synthesize the data, from which the sample size of a definitive RCT can be estimated."

METHODS

3a) Description of trial design (such as parallel, factorial) including allocation ratio

Does your paper address CONSORT subitem 3a? *

Copy and paste relevant sections from the manuscript (include quotes in quotation marks "like this" to indicate direct quotes from your manuscript), or elaborate on this item by providing additional information not in the ms, or briefly explain why the item is not applicable/relevant for your study

"A pilot or feasibility study is intended to guide the planning of a large-scale investigation. "

3b) Important changes to methods after trial commencement (such as eligibility criteria), with reasons

Does your paper address CONSORT subitem 3b? *

Copy and paste relevant sections from the manuscript (include quotes in quotation marks "like this" to indicate direct quotes from your manuscript), or elaborate on this item by providing additional information not in the ms, or briefly explain why the item is not applicable/relevant for your study

"The trial protocol originally proposed to include outpatient adults with three specific diagnoses.[5] However, during the intervention sessions, therapists using the FEPSim device identified eight additional diagnoses that could potentially benefit from its use. As a result, we amended the trial protocol to include patients with a total of 11 diagnoses, as outlined in the methods section."

3b-i) Bug fixes, Downtimes, Content Changes

Bug fixes, Downtimes, Content Changes: ehealth systems are often dynamic systems. A description of changes to methods therefore also includes important changes made on the intervention or comparator during the trial (e.g., major bug fixes or changes in the functionality or content) (5-iii) and other "unexpected events" that may have influenced study design such as staff changes, system failures/downtimes, etc. [2].

	1	2	3	4	5	
subitem not at all important	◉	○	○	○	○	essential

Clear selection

Does your paper address subitem 3b-i?

Copy and paste relevant sections from the manuscript (include quotes in quotation marks "like this" to indicate direct quotes from your manuscript), or elaborate on this item by providing additional information not in the ms, or briefly explain why the item is not applicable/relevant for your study

Not applicable as this is not a mobile APP intervention

4a) Eligibility criteria for participants

Does your paper address CONSORT subitem 4a? *

Copy and paste relevant sections from the manuscript (include quotes in quotation marks "like this" to indicate direct quotes from your manuscript), or elaborate on this item by providing additional information not in the ms, or briefly explain why the item is not applicable/relevant for your study

"The participants were outpatient adults (18 years of age and older) with limitations in their forearm, wrist, or hand function due to distal radial/ulnar fractures, stroke, or osteoarthritis (i.e., patients who had undergone wrist salvage surgery), Keinbock's disease, ulnar shortening, perilunates dislocation, ganglion removal, tumor removals, triangular fibrocartilage complex injury repairs, ligament repair (torn ligament), and spinal cord injury. The study excluded patients with chronic regional pain syndrome (CRPS), those who experienced unmanaged pain or edema, individuals with communication or cognitive impairments that hindered their ability to understand instructions, and those who were not able to participate in the outpatient hand program at least once a week. "

4a-i) Computer / Internet literacy

Computer / Internet literacy is often an implicit “de facto” eligibility criterion - this should be explicitly clarified.

	1	2	3	4	5	
subitem not at all important	(selected)	○	○	○	○	essential

Clear selection

Does your paper address subitem 4a-i?

Copy and paste relevant sections from the manuscript (include quotes in quotation marks "like this" to indicate direct quotes from your manuscript), or elaborate on this item by providing additional information not in the ms, or briefly explain why the item is not applicable/relevant for your study

Not applicable as this is not a mobile APP intervention

4a-ii) Open vs. closed, web-based vs. face-to-face assessments:

Open vs. closed, web-based vs. face-to-face assessments: Mention how participants were recruited (online vs. offline), e.g., from an open access website or from a clinic, and clarify if this was a purely web-based trial, or there were face-to-face components (as part of the intervention or for assessment), i.e., to what degree got the study team to know the participant. In online-only trials, clarify if participants were quasi-anonymous and whether having multiple identities was possible or whether technical or logistical measures (e.g., cookies, email confirmation, phone calls) were used to detect/prevent these.

	1	2	3	4	5	
subitem not at all important	○	○	○	○	◉	essential

Clear selection

Does your paper address subitem 4a-ii? *

Copy and paste relevant sections from the manuscript (include quotes in quotation marks "like this" to indicate direct quotes from your manuscript), or elaborate on this item by providing additional information not in the ms, or briefly explain why the item is not applicable/relevant for your study

"Patients receiving outpatient hand therapy at the Glenrose Rehabilitation Hospital and Royal Alexandra Hospital (Alberta Health Services) who met the inclusion criteria were invited to participate in the trial. "

4a-iii) Information giving during recruitment

Information given during recruitment. Specify how participants were briefed for recruitment and in the informed consent procedures (e.g., publish the informed consent documentation as appendix, see also item X26), as this information may have an effect on user self-selection, user expectation and may also bias results.

	1	2	3	4	5	
subitem not at all important	○	○	○	○	◉	essential

Clear selection

Does your paper address subitem 4a-iii?

Copy and paste relevant sections from the manuscript (include quotes in quotation marks "like this" to indicate direct quotes from your manuscript), or elaborate on this item by providing additional information not in the ms, or briefly explain why the item is not applicable/relevant for your study

"Then, the participants who met inclusion criteria and consented to participate were allocated into one of the groups (experimental group or control group) by the project coordinator who was off-site. This was a single-blinded study. Research assistants blinded to group allocation collected demographics (age, gender, ethnicity, employment status, highest degree or level of education and medication taken) and outcome measures. Outcome measures were collected at enrollment and weeks two, four, eight and ten."

4b) Settings and locations where the data were collected

Does your paper address CONSORT subitem 4b? *

Copy and paste relevant sections from the manuscript (include quotes in quotation marks "like this" to indicate direct quotes from your manuscript), or elaborate on this item by providing additional information not in the ms, or briefly explain why the item is not applicable/relevant for your study

"Patients receiving outpatient hand therapy at the Glenrose Rehabilitation Hospital and Royal Alexandra Hospital (Alberta Health Services) who met the inclusion criteria were invited to participate in the trial. "

4b-i) Report if outcomes were (self-)assessed through online questionnaires

Clearly report if outcomes were (self-)assessed through online questionnaires (as common in web-based trials) or otherwise.

	1	2	3	4	5	
subitem not at all important	(●)	○	○	○	○	essential

Clear selection

Does your paper address subitem 4b-i? *

Copy and paste relevant sections from the manuscript (include quotes in quotation marks "like this" to indicate direct quotes from your manuscript), or elaborate on this item by providing additional information not in the ms, or briefly explain why the item is not applicable/relevant for your study

Not applicable as this is not a mobile APP intervention

4b-ii) Report how institutional affiliations are displayed

Report how institutional affiliations are displayed to potential participants [on ehealth media], as affiliations with prestigious hospitals or universities may affect volunteer rates, use, and reactions with regards to an intervention.(Not a required item – describe only if this may bias results)

	1	2	3	4	5	
subitem not at all important	○	○	○	○	◉	essential

Clear selection

Does your paper address subitem 4b-ii?

Copy and paste relevant sections from the manuscript (include quotes in quotation marks "like this" to indicate direct quotes from your manuscript), or elaborate on this item by providing additional information not in the ms, or briefly explain why the item is not applicable/relevant for your study

"Patients receiving outpatient hand therapy at the Glenrose Rehabilitation Hospital and Royal Alexandra Hospital (Alberta Health Services) who met the inclusion criteria were invited to participate in the trial. "

5) The interventions for each group with sufficient details to allow replication, including how and when they were actually administered

5-i) Mention names, credential, affiliations of the developers, sponsors, and owners

Mention names, credential, affiliations of the developers, sponsors, and owners [6] (if authors/evaluators are owners or developer of the software, this needs to be declared in a “Conflict of interest” section or mentioned elsewhere in the manuscript).

	1	2	3	4	5	
subitem not at all important	○	○	○	○	◉	essential

Clear selection

Does your paper address subitem 5-i?

Copy and paste relevant sections from the manuscript (include quotes in quotation marks "like this" to indicate direct quotes from your manuscript), or elaborate on this item by providing additional information not in the ms, or briefly explain why the item is not applicable/relevant for your study

"The FEPSim®, developed by Karma Machining & Manufacturing Ltd, is a rehabilitation device designed explicitly for hand therapy. "

5-ii) Describe the history/development process

Describe the history/development process of the application and previous formative evaluations (e.g., focus groups, usability testing), as these will have an impact on adoption/use rates and help with interpreting results.

	1	2	3	4	5	
subitem not at all important	○	○	○	○	◉	essential

Clear selection

Does your paper address subitem 5-ii?

Copy and paste relevant sections from the manuscript (include quotes in quotation marks "like this" to indicate direct quotes from your manuscript), or elaborate on this item by providing additional information not in the ms, or briefly explain why the item is not applicable/relevant for your study

"The FEPSim®, developed by Karma Machining & Manufacturing Ltd, is a rehabilitation device designed explicitly for hand therapy. It targets hand and wrist conditioning through various movements and offers adaptability for different grasp patterns. The device allows therapists to monitor improvements in strength, range of motion, and endurance by enabling controlled resistance adjustments. Resistance is set using a lever on a generic scale from 0 to 1, which corresponds to a torque range of 0.1 to 3.7 Newton-meters. In addition to resistance, the device can record the repetitions of therapeutic exercises, and is equipped with degree scales that allow therapists to observe patients' range of motion during active movements of the hand, wrist, or forearm. Thus, although the FEPSim® is not an assessment tool, it serves as a useful tool for tracking patient progress in hand therapy. Its potential advantages also include compactness and portability, making it a promising option for rehabilitation of impairments of the hand, wrist, and forearm; however, the effect of using the FEPSim® on hand therapy outcomes is unknown. In addition, the device, priced at less than US $3,000."

5-iii) Revisions and updating

Revisions and updating. Clearly mention the date and/or version number of the application/intervention (and comparator, if applicable) evaluated, or describe whether the intervention underwent major changes during the evaluation process, or whether the development and/or content was “frozen” during the trial. Describe dynamic components such as news feeds or changing content which may have an impact on the replicability of the intervention (for unexpected events see item 3b).

	1	2	3	4	5	
subitem not at all important	○	○	○	○	◉	essential

Clear selection

Does your paper address subitem 5-iii?

Copy and paste relevant sections from the manuscript (include quotes in quotation marks "like this" to indicate direct quotes from your manuscript), or elaborate on this item by providing additional information not in the ms, or briefly explain why the item is not applicable/relevant for your study

"The study employed the horizontal model depicted in Figure 1. This device features a central structure with an adjustable resistance mechanism, a degree-of-rotation scale for supination and pronation, and a small screen displaying exercise repetition counts. The device is tabletop secure and comprises two lateral shafts."

5-iv) Quality assurance methods

Provide information on quality assurance methods to ensure accuracy and quality of information provided [1], if applicable.

	1	2	3	4	5	
subitem not at all important	○	○	○	○	◉	essential

Clear selection

Does your paper address subitem 5-iv?

Copy and paste relevant sections from the manuscript (include quotes in quotation marks "like this" to indicate direct quotes from your manuscript), or elaborate on this item by providing additional information not in the ms, or briefly explain why the item is not applicable/relevant for your study

"The study employed the horizontal model depicted in Figure 1. This device features a central structure with an adjustable resistance mechanism, a degree-of-rotation scale for supination and pronation, and a small screen displaying exercise repetition counts. The device is tabletop secure and comprises two lateral shafts.
Various attachments facilitate exercises and the simulation of daily activities. For instance, a dowel grip attachment, installable on each shaft, is utilized for wrist flexion and extension exercises. Rotation and supination exercises involve a 90-degree unit rotation, enabling patients to grasp the dowel and perform clockwise and counter-clockwise rotations.
Additional attachments include a key pinch attachment for lateral pinch exercises resembling tasks like starting a car or turning a key, a T attachment simulating can opener use, a lever attachment for elbow extension/flexion exercises and simulating knife cutting and steering wheel use, and a doorknob attachment for door opening simulations.
"

5-v) Ensure replicability by publishing the source code, and/or providing screenshots/screen-capture video, and/or providing flowcharts of the algorithms used

Ensure replicability by publishing the source code, and/or providing screenshots/screen-capture video, and/or providing flowcharts of the algorithms used. Replicability (i.e., other researchers should in principle be able to replicate the study) is a hallmark of scientific reporting.

	1	2	3	4	5	
subitem not at all important	○	○	○	○	◉	essential

Clear selection

Does your paper address subitem 5-v?

Copy and paste relevant sections from the manuscript (include quotes in quotation marks "like this" to indicate direct quotes from your manuscript), or elaborate on this item by providing additional information not in the ms, or briefly explain why the item is not applicable/relevant for your study

"The study employed the horizontal model depicted in Figure 1. This device features a central structure with an adjustable resistance mechanism, a degree-of-rotation scale for supination and pronation, and a small screen displaying exercise repetition counts. The device is tabletop secure and comprises two lateral shafts.
Various attachments facilitate exercises and the simulation of daily activities. For instance, a dowel grip attachment, installable on each shaft, is utilized for wrist flexion and extension exercises. Rotation and supination exercises involve a 90-degree unit rotation, enabling patients to grasp the dowel and perform clockwise and counter-clockwise rotations.
Additional attachments include a key pinch attachment for lateral pinch exercises resembling tasks like starting a car or turning a key, a T attachment simulating can opener use, a lever attachment for elbow extension/flexion exercises and simulating knife cutting and steering wheel use, and a doorknob attachment for door opening simulations.
"

5-vi) Digital preservation

Digital preservation: Provide the URL of the application, but as the intervention is likely to change or disappear over the course of the years; also make sure the intervention is archived (Internet Archive, webcitation.org, and/or publishing the source code or screenshots/videos alongside the article). As pages behind login screens cannot be archived, consider creating demo pages which are accessible without login.

	1	2	3	4	5	
subitem not at all important	◉	○	○	○	○	essential

Clear selection

Does your paper address subitem 5-vi?

Copy and paste relevant sections from the manuscript (include quotes in quotation marks "like this" to indicate direct quotes from your manuscript), or elaborate on this item by providing additional information not in the ms, or briefly explain why the item is not applicable/relevant for your study

Not applicable as this is not a mobile APP intervention

5-vii) Access

Access: Describe how participants accessed the application, in what setting/context, if they had to pay (or were paid) or not, whether they had to be a member of specific group. If known, describe how participants obtained "access to the platform and Internet" [1]. To ensure access for editors/reviewers/readers, consider to provide a "backdoor" login account or demo mode for reviewers/readers to explore the application (also important for archiving purposes, see vi).

	1	2	3	4	5	
subitem not at all important	○	○	○	○	●	essential

Clear selection

Does your paper address subitem 5-vii? *

Copy and paste relevant sections from the manuscript (include quotes in quotation marks "like this" to indicate direct quotes from your manuscript), or elaborate on this item by providing additional information not in the ms, or briefly explain why the item is not applicable/relevant for your study

"The FEPSim®: A novel device for rehabilitation of the hand, wrist, and forearm (https://fepsim.com/)"

5-viii) Mode of delivery, features/functionalities/components of the intervention and comparator, and the theoretical framework

Describe mode of delivery, features/functionalities/components of the intervention and comparator, and the theoretical framework [6] used to design them (instructional strategy [1], behaviour change techniques, persuasive features, etc., see e.g., [7, 8] for terminology). This includes an in-depth description of the content (including where it is coming from and who developed it) [1],” whether [and how] it is tailored to individual circumstances and allows users to track their progress and receive feedback” [6]. This also includes a description of communication delivery channels and – if computer-mediated communication is a component – whether communication was synchronous or asynchronous [6]. It also includes information on presentation strategies [1], including page design principles, average amount of text on pages, presence of hyperlinks to other resources, etc. [1].

	1	2	3	4	5	
subitem not at all important	○	○	○	○	◉	essential

Clear selection

Does your paper address subitem 5-viii? *

Copy and paste relevant sections from the manuscript (include quotes in quotation marks "like this" to indicate direct quotes from your manuscript), or elaborate on this item by providing additional information not in the ms, or briefly explain why the item is not applicable/relevant for your study

"The study employed the horizontal model depicted in Figure 1. This device features a central structure with an adjustable resistance mechanism, a degree-of-rotation scale for supination and pronation, and a small screen displaying exercise repetition counts. The device is tabletop secure and comprises two lateral shafts.
Various attachments facilitate exercises and the simulation of daily activities. For instance, a dowel grip attachment, installable on each shaft, is utilized for wrist flexion and extension exercises. Rotation and supination exercises involve a 90-degree unit rotation, enabling patients to grasp the dowel and perform clockwise and counter-clockwise rotations. Additional attachments include a key pinch attachment for lateral pinch exercises resembling tasks like starting a car or turning a key, a T attachment simulating can opener use, a lever attachment for elbow extension/flexion exercises and simulating knife cutting and steering wheel use, and a doorknob attachment for door opening simulations.
"

5-ix) Describe use parameters

Describe use parameters (e.g., intended “doses” and optimal timing for use). Clarify what instructions or recommendations were given to the user, e.g., regarding timing, frequency, heaviness of use, if any, or was the intervention used ad libitum.

	1	2	3	4	5	
subitem not at all important	○	○	○	○	◉	essential

Clear selection

Does your paper address subitem 5-ix?

Copy and paste relevant sections from the manuscript (include quotes in quotation marks "like this" to indicate direct quotes from your manuscript), or elaborate on this item by providing additional information not in the ms, or briefly explain why the item is not applicable/relevant for your study

"Both groups received standard care at each hospital, which consisted of immobilization for seven to eight weeks after the time of the injury or surgery (if needed), followed by hand therapy sessions for about ten weeks. Hand therapy sessions included the management of scar tissue, sensory alterations, and edema, and therapeutic activities to increase strength, range of motion, resistance, and dexterity. For both groups the sessions’ length and frequency depended on the patients’ individualized needs and diagnoses, as determined by their treating therapists. The length of each session was between 30 and 45 minutes, carried out once or twice per week. The sessions were provided by occupational or physical therapists with training and experience in hand therapy."

5-x) Clarify the level of human involvement

Clarify the level of human involvement (care providers or health professionals, also technical assistance) in the e-intervention or as co-intervention (detail number and expertise of professionals involved, if any, as well as “type of assistance offered, the timing and frequency of the support, how it is initiated, and the medium by which the assistance is delivered”. It may be necessary to distinguish between the level of human involvement required for the trial, and the level of human involvement required for a routine application outside of a RCT setting (discuss under item 21 – generalizability).

	1	2	3	4	5	
subitem not at all important	○	○	○	○	◉	essential

Clear selection

Does your paper address subitem 5-x?

Copy and paste relevant sections from the manuscript (include quotes in quotation marks "like this" to indicate direct quotes from your manuscript), or elaborate on this item by providing additional information not in the ms, or briefly explain why the item is not applicable/relevant for your study

"The sessions were provided by occupational or physical therapists with training and experience in hand therapy."

5-xi) Report any prompts/reminders used

Report any prompts/reminders used: Clarify if there were prompts (letters, emails, phone calls, SMS) to use the application, what triggered them, frequency etc. It may be necessary to distinguish between the level of prompts/reminders required for the trial, and the level of prompts/reminders for a routine application outside of a RCT setting (discuss under item 21 – generalizability).

	1	2	3	4	5	
subitem not at all important	◉	○	○	○	○	essential

Clear selection

Does your paper address subitem 5-xi? *

Copy and paste relevant sections from the manuscript (include quotes in quotation marks "like this" to indicate direct quotes from your manuscript), or elaborate on this item by providing additional information not in the ms, or briefly explain why the item is not applicable/relevant for your study

Not applicable as this is not a mobile APP intervention

5-xii) Describe any co-interventions (incl. training/support)

Describe any co-interventions (incl. training/support): Clearly state any interventions that are provided in addition to the targeted eHealth intervention, as ehealth intervention may not be designed as stand-alone intervention. This includes training sessions and support [1]. It may be necessary to distinguish between the level of training required for the trial, and the level of training for a routine application outside of a RCT setting (discuss under item 21 – generalizability.

	1	2	3	4	5	
subitem not at all important	◉	○	○	○	○	essential

Clear selection

Does your paper address subitem 5-xii? *

Copy and paste relevant sections from the manuscript (include quotes in quotation marks "like this" to indicate direct quotes from your manuscript), or elaborate on this item by providing additional information not in the ms, or briefly explain why the item is not applicable/relevant for your study

Not applicable as this is not a mobile APP intervention

6a) Completely defined pre-specified primary and secondary outcome measures, including how and when they were assessed

Does your paper address CONSORT subitem 6a? *

Copy and paste relevant sections from the manuscript (include quotes in quotation marks "like this" to indicate direct quotes from your manuscript), or elaborate on this item by providing additional information not in the ms, or briefly explain why the item is not applicable/relevant for your study

"The primary outcome measures were range of motion and grip strength. Range of motion included active range of motion (AROM) and passive range of motion (PROM) of the wrist extension, flexion, and radial and ulnar deviation; pronation and supination were measured using a 12-inch clear plastic goniometer (Baseline 360-degree); grip strength was measured using a hydraulic dynamometer (Baseline Lite hydraulic, 200 lb); pinch strength (including pulp pinch, key grip, three finger grip) was measured using a pinch gauge or pinch meter – 60 lb. Grip and pinch strength were measured three times, then the mean and standard deviation of the three measures were calculated. The secondary outcome variables were the patients' perceived wrist pain and disability in activities of daily living and was measured with the patient-rated wrist evaluation questionnaire (PRWE). The PRWE is a valid and reliable assessment tool of patient-based pain and disability in three components: pain; function during specific activities of daily living; and functioning during usual activities (personal care, household work, work, and recreational activities).[5] Each item is rated from 1-10 and the maximum PRWE score is 100. Lower values in the PRWHE scale outcome variable are desirable with hand therapy interventions." "Research assistants blinded to group allocation collected demographics (age, gender, ethnicity, employment status, highest degree or level of education and medication taken) and outcome measures. Outcome measures were collected at enrollment and weeks two, four, eight and ten."

6a-i) Online questionnaires: describe if they were validated for online use and apply CHERRIES items to describe how the questionnaires were designed/deployed

If outcomes were obtained through online questionnaires, describe if they were validated for online use and apply CHERRIES items to describe how the questionnaires were designed/deployed [9].

	1	2	3	4	5	
subitem not at all important	◉	○	○	○	○	essential

Clear selection

Does your paper address subitem 6a-i?

Copy and paste relevant sections from manuscript text

Not applicable as this is not a mobile APP intervention

6a-ii) Describe whether and how “use” (including intensity of use/dosage) was defined/measured/monitored

Describe whether and how “use” (including intensity of use/dosage) was defined/measured/monitored (logins, logfile analysis, etc.). Use/adoption metrics are important process outcomes that should be reported in any ehealth trial.

	1	2	3	4	5	
subitem not at all important	○	○	○	○	◉	essential

Clear selection

Does your paper address subitem 6a-ii?

Copy and paste relevant sections from manuscript text

"For both groups the sessions’ length and frequency depended on the patients’ individualized needs and diagnoses, as determined by their treating therapists. The length of each session was between 30 and 45 minutes, carried out once or twice per week. The sessions were provided by occupational or physical therapists with training and experience in hand therapy."

6a-iii) Describe whether, how, and when qualitative feedback from participants was obtained

Describe whether, how, and when qualitative feedback from participants was obtained (e.g., through emails, feedback forms, interviews, focus groups).

	1	2	3	4	5	
subitem not at all important	○	○	○	○	◉	essential

Clear selection

Does your paper address subitem 6a-iii?

Copy and paste relevant sections from manuscript text

"The therapists' acceptance of this device is also backed up by the qualitative results of another study published elsewhere.[14]"

6b) Any changes to trial outcomes after the trial commenced, with reasons

Does your paper address CONSORT subitem 6b? *

Copy and paste relevant sections from the manuscript (include quotes in quotation marks "like this" to indicate direct quotes from your manuscript), or elaborate on this item by providing additional information not in the ms, or briefly explain why the item is not applicable/relevant for your study

No changes in outcome measures

7a) How sample size was determined

NPT: When applicable, details of whether and how the clustering by care provides or centers was addressed

7a-i) Describe whether and how expected attrition was taken into account when calculating the sample size

Describe whether and how expected attrition was taken into account when calculating the sample size.

	1	2	3	4	5	
subitem not at all important	○	○	○	○	◉	essential

Clear selection

Does your paper address subitem 7a-i?

Copy and paste relevant sections from manuscript title (include quotes in quotation marks "like this" to indicate direct quotes from your manuscript), or elaborate on this item by providing additional information not in the ms, or briefly explain why the item is not applicable/relevant for your study

"The trial protocol originally proposed to include outpatient adults with three specific diagnoses.[5] However, during the intervention sessions, therapists using the FEPSim® device identified eight additional diagnoses that could potentially benefit from its use. As a result, we amended the trial protocol to include patients with a total of 11 diagnoses, as outlined in the methods section."

7b) When applicable, explanation of any interim analyses and stopping guidelines

Does your paper address CONSORT subitem 7b? *

Copy and paste relevant sections from the manuscript (include quotes in quotation marks "like this" to indicate direct quotes from your manuscript), or elaborate on this item by providing additional information not in the ms, or briefly explain why the item is not applicable/relevant for your study

No interim analyses and stopping guidelines were in place

8a) Method used to generate the random allocation sequence
NPT: When applicable, how care providers were allocated to each trial group

Does your paper address CONSORT subitem 8a? *

Copy and paste relevant sections from the manuscript (include quotes in quotation marks "like this" to indicate direct quotes from your manuscript), or elaborate on this item by providing additional information not in the ms, or briefly explain why the item is not applicable/relevant for your study

"Hand therapists at both hospitals who were unaware of the randomization blocks identified eligible participants. Then, the participants who met inclusion criteria and consented to participate were allocated into one of the groups (experimental group or control group) by the project coordinator who was off-site. "

8b) Type of randomisation; details of any restriction (such as blocking and block size)

Does your paper address CONSORT subitem 8b? *

Copy and paste relevant sections from the manuscript (include quotes in quotation marks "like this" to indicate direct quotes from your manuscript), or elaborate on this item by providing additional information not in the ms, or briefly explain why the item is not applicable/relevant for your study

"Hand therapists at both hospitals who were unaware of the randomization blocks identified eligible participants. Then, the participants who met inclusion criteria and consented to participate were allocated into one of the groups (experimental group or control group) by the project coordinator who was off-site. "

9) Mechanism used to implement the random allocation sequence (such as sequentially numbered containers), describing any steps taken to conceal the sequence until interventions were assigned

Does your paper address CONSORT subitem 9? *

Copy and paste relevant sections from the manuscript (include quotes in quotation marks "like this" to indicate direct quotes from your manuscript), or elaborate on this item by providing additional information not in the ms, or briefly explain why the item is not applicable/relevant for your study

"Hand therapists at both hospitals who were unaware of the randomization blocks identified eligible participants. Then, the participants who met inclusion criteria and consented to participate were allocated into one of the groups (experimental group or control group) by the project coordinator who was off-site. "

10) Who generated the random allocation sequence, who enrolled participants, and who assigned participants to interventions

Does your paper address CONSORT subitem 10? *

Copy and paste relevant sections from the manuscript (include quotes in quotation marks "like this" to indicate direct quotes from your manuscript), or elaborate on this item by providing additional information not in the ms, or briefly explain why the item is not applicable/relevant for your study

"Hand therapists at both hospitals who were unaware of the randomization blocks identified eligible participants. Then, the participants who met inclusion criteria and consented to participate were allocated into one of the groups (experimental group or control group) by the project coordinator who was off-site. "

11a) If done, who was blinded after assignment to interventions (for example, participants, care providers, those assessing outcomes) and how

NPT: Whether or not administering co-interventions were blinded to group assignment

11a-i) Specify who was blinded, and who wasn’t

Specify who was blinded, and who wasn’t. Usually, in web-based trials it is not possible to blind the participants [1, 3] (this should be clearly acknowledged), but it may be possible to blind outcome assessors, those doing data analysis or those administering co-interventions (if any).

	1	2	3	4	5	
subitem not at all important	○	○	○	○	◉	essential

Clear selection

Does your paper address subitem 11a-i? *

Copy and paste relevant sections from the manuscript (include quotes in quotation marks "like this" to indicate direct quotes from your manuscript), or elaborate on this item by providing additional information not in the ms, or briefly explain why the item is not applicable/relevant for your study

"This was a single-blinded study. Research assistants blinded to group allocation collected demographics (age, gender, ethnicity, employment status, highest degree or level of education and medication taken) and outcome measures. Outcome measures were collected at enrollment and weeks two, four, eight and ten."

11a-ii) Discuss e.g., whether participants knew which intervention was the “intervention of interest” and which one was the “comparator”

Informed consent procedures (4a-ii) can create biases and certain expectations - discuss e.g., whether participants knew which intervention was the “intervention of interest” and which one was the “comparator”.

	1	2	3	4	5	
subitem not at all important	○	○	○	○	◉	essential

Clear selection

Does your paper address subitem 11a-ii?

Copy and paste relevant sections from the manuscript (include quotes in quotation marks "like this" to indicate direct quotes from your manuscript), or elaborate on this item by providing additional information not in the ms, or briefly explain why the item is not applicable/relevant for your study

"Hand therapists at both hospitals who were unaware of the randomization blocks identified eligible participants. Then, the participants who met inclusion criteria and consented to participate were allocated into one of the groups (experimental group or control group) by the project coordinator who was off-site. This was a single-blinded study. Research assistants blinded to group allocation collected demographics (age, gender, ethnicity, employment status, highest degree or level of education and medication taken) and outcome measures. Outcome measures were collected at enrollment and weeks two, four, eight and ten."

11b) If relevant, description of the similarity of interventions

(this item is usually not relevant for ehealth trials as it refers to similarity of a placebo or sham intervention to a active medication/intervention)

Does your paper address CONSORT subitem 11b? *

Copy and paste relevant sections from the manuscript (include quotes in quotation marks "like this" to indicate direct quotes from your manuscript), or elaborate on this item by providing additional information not in the ms, or briefly explain why the item is not applicable/relevant for your study

"2. High-cost Electromechanical Devices: This category includes both portable and non-portable commercial electromechanical devices whose cost ranges from US $10,000 to US $95,000. Examples are the Baltimore Therapeutic Equipment (BTE) work simulator and SaeboReJoyce. The BTE has been shown to be a reliable and valid tool, comparable to standard dynamometers, for measuring grip strength in healthy individuals [6,7]. The SaeboReJoyce provides a valid, quantitative, and automated alternative to standardized hand function assessments especially for patients with stroke [8]; however, its effectiveness in improving upper limb function remains inconclusive [9]. "

12a) Statistical methods used to compare groups for primary and secondary outcomes

NPT: When applicable, details of whether and how the clustering by care providers or centers was addressed

Does your paper address CONSORT subitem 12a? *

Copy and paste relevant sections from the manuscript (include quotes in quotation marks "like this" to indicate direct quotes from your manuscript), or elaborate on this item by providing additional information not in the ms, or briefly explain why the item is not applicable/relevant for your study

"The analysis was conducted using an intention-to-treat principle. Descriptive statistics were used to characterize the groups at the pre- and post-test. Due to variations in the lengths of the interventions, as a result of various discharge times from treatment services, post-test was defined as the last measure taken once discharge from the outpatient clinic was determined by the hand therapists. Tests of normality were carried out in within and between groups comparisons across the outcome variables as appropriate (i.e., paired t-test or Wilcoxon signed-ranks test for within group comparisons; independent t-test or Mann-Whitney U test for between group comparisons). We calculated 95% confidence intervals and interpreted the level of uncertainty based on them.[12] The alpha level of significance was set at p < .05 (one-tailed). Cohen's d values were calculated across variables, and an overall average effect size was calculated using a fixed-effects model for continuous outcomes. All analyses were conducted using SPSS (Version 28, Armonk, NY, IBM Corp.) In addition, the minimal clinically important difference (MCID) was calculated. An effect size of 0.2 is commonly considered equivalent to the MCID, so we estimated it by multiplying the standard deviation of the baseline scores by 0.2 (a small effect size) [13]."

12a-i) Imputation techniques to deal with attrition / missing values

Imputation techniques to deal with attrition / missing values: Not all participants will use the intervention/comparator as intended and attrition is typically high in ehealth trials. Specify how participants who did not use the application or dropped out from the trial were treated in the statistical analysis (a complete case analysis is strongly discouraged, and simple imputation techniques such as LOCF may also be problematic [4]).

	1	2	3	4	5	
subitem not at all important	○	○	○	○	◉	essential

Clear selection

Does your paper address subitem 12a-i? *

Copy and paste relevant sections from the manuscript (include quotes in quotation marks "like this" to indicate direct quotes from your manuscript), or elaborate on this item by providing additional information not in the ms, or briefly explain why the item is not applicable/relevant for your study

"Another important consideration is that the two variables showing statistically significant differences between groups—grip strength and passive wrist flexion—had missing values in 19% and 14% of participants, respectively. These missing pretest values were imputed using the single imputation method of carrying the last observation backward. While this approach is widely used, it, like all imputation methods, has inherent limitations. "

12b) Methods for additional analyses, such as subgroup analyses and adjusted analyses

Does your paper address CONSORT subitem 12b? *

Copy and paste relevant sections from the manuscript (include quotes in quotation marks "like this" to indicate direct quotes from your manuscript), or elaborate on this item by providing additional information not in the ms, or briefly explain why the item is not applicable/relevant for your study

"In addition, the minimal clinically important difference (MCID) was calculated. An effect size of 0.2 is commonly considered equivalent to the MCID, so we estimated it by multiplying the standard deviation of the baseline scores by 0.2 (a small effect size) [13]. "

X26) REB/IRB Approval and Ethical Considerations [recommended as subheading under "Methods"] (not a CONSORT item)

X26-i) Comment on ethics committee approval

	1	2	3	4	5	
subitem not at all important	○	○	○	○	◉	essential

Clear selection

Does your paper address subitem X26-i?

Copy and paste relevant sections from the manuscript (include quotes in quotation marks "like this" to indicate direct quotes from your manuscript), or elaborate on this item by providing additional information not in the ms, or briefly explain why the item is not applicable/relevant for your study

"The University of Alberta Ethics Board (Pro00095587) and the Northern Alberta Clinical Trials Research Centre. The trial was registered in the International Standard Randomized Controlled Trial Number (ISRCTN13656014)."

x26-ii) Outline informed consent procedures

Outline informed consent procedures e.g., if consent was obtained offline or online (how? Checkbox, etc.?), and what information was provided (see 4a-ii). See [6] for some items to be included in informed consent documents.

	1	2	3	4	5	
subitem not at all important	○	○	○	○	◉	essential

Clear selection

Does your paper address subitem X26-ii?

Copy and paste relevant sections from the manuscript (include quotes in quotation marks "like this" to indicate direct quotes from your manuscript), or elaborate on this item by providing additional information not in the ms, or briefly explain why the item is not applicable/relevant for your study

"Invitations to participate were posted at several locations in the two hospitals. Hand therapists at both hospitals who were unaware of the randomization blocks identified eligible participants. Then, the participants who met inclusion criteria and consented to participate were allocated into one of the groups (experimental group or control group) by the project coordinator who was off-site. "

X26-iii) Safety and security procedures

Safety and security procedures, incl. privacy considerations, and any steps taken to reduce the likelihood or detection of harm (e.g., education and training, availability of a hotline)

	1	2	3	4	5	
subitem not at all important	○	○	○	○	◉	essential

Clear selection

Does your paper address subitem X26-iii?

Copy and paste relevant sections from the manuscript (include quotes in quotation marks "like this" to indicate direct quotes from your manuscript), or elaborate on this item by providing additional information not in the ms, or briefly explain why the item is not applicable/relevant for your study

"The sessions were provided by occupational or physical therapists with training and experience in hand therapy."

RESULTS

13a) For each group, the numbers of participants who were randomly assigned, received intended treatment, and were analysed for the primary outcome

NPT: The number of care providers or centers performing the intervention in each group and the number of patients treated by each care provider in each center

Does your paper address CONSORT subitem 13a? *

Copy and paste relevant sections from the manuscript (include quotes in quotation marks "like this" to indicate direct quotes from your manuscript), or elaborate on this item by providing additional information not in the ms, or briefly explain why the item is not applicable/relevant for your study

"From October 1, 2020 to August 31, 2022, 7,160 patients received hand therapy rehabilitation at the two hospitals, from which 110 were eligible to enter the trial and 38 consented to participate (participation rate of 35%). The trial retention rate was 95% (36/38). The retention rate for the control group was 100% and for the intervention group it was 89% (17/19). Two participants declined to participate after the first assessment due to disagreement with measures in place to control the spread of the pandemic. Figure 2 presents the flow diagram of the progress during the phases of this study. Table 1 presents the demographics of the 36 participants who completed the study and table 2 presents the participants' baseline characteristics according to group allocation.

Figure 2. Consolidated Standards of Reporting Trials flow diagram"

13b) For each group, losses and exclusions after randomisation, together with reasons

Does your paper address CONSORT subitem 13b? (NOTE: Preferably, this is shown in a CONSORT flow diagram) *

Copy and paste relevant sections from the manuscript (include quotes in quotation marks "like this" to indicate direct quotes from your manuscript), or elaborate on this item by providing additional information not in the ms, or briefly explain why the item is not applicable/relevant for your study

"Two participants declined to participate after the first assessment due to disagreement with measures in place to control the spread of the pandemic. "

13b-i) Attrition diagram

Strongly recommended: An attrition diagram (e.g., proportion of participants still logging in or using the intervention/comparator in each group plotted over time, similar to a survival curve) or other figures or tables demonstrating usage/dose/engagement.

	1	2	3	4	5	
subitem not at all important	○	○	○	○	◉	essential

Clear selection

Does your paper address subitem 13b-i?

Copy and paste relevant sections from the manuscript or cite the figure number if applicable (include quotes in quotation marks "like this" to indicate direct quotes from your manuscript), or elaborate on this item by providing additional information not in the ms, or briefly explain why the item is not applicable/relevant for your study

" Figure 2 presents the flow diagram of the progress during the phases of this study. Table 1 presents the demographics of the 36 participants who completed the study and table 2 presents the participants' baseline characteristics according to group allocation.

Figure 2. Consolidated Standards of Reporting Trials flow diagram"

14a) Dates defining the periods of recruitment and follow-up

Does your paper address CONSORT subitem 14a? *

Copy and paste relevant sections from the manuscript (include quotes in quotation marks "like this" to indicate direct quotes from your manuscript), or elaborate on this item by providing additional information not in the ms, or briefly explain why the item is not applicable/relevant for your study

"From October 1, 2020 to August 31, 2022, 7,160 patients received hand therapy rehabilitation at the two hospitals, from which 110 were eligible to enter the trial and 38 consented to participate (participation rate of 35%). "

14a-i) Indicate if critical “secular events” fell into the study period

Indicate if critical “secular events” fell into the study period, e.g., significant changes in Internet resources available or “changes in computer hardware or Internet delivery resources”

	1	2	3	4	5	
subitem not at all important	○	○	○	○	◉	essential

Clear selection

Does your paper address subitem 14a-i?

Copy and paste relevant sections from the manuscript (include quotes in quotation marks "like this" to indicate direct quotes from your manuscript), or elaborate on this item by providing additional information not in the ms, or briefly explain why the item is not applicable/relevant for your study

"The COVID-19 pandemic had a significant impact on the trial, with different hospital policies for COVID-19 prevention and control, resulting in varying recruitment launch dates and the closure of one hospital's hand therapy service. This led to a significant reduction in the number of potential participants who met the inclusion criteria. In addition, the data collection was affected, as the trial was conducted over several months during the pandemic."

14b) Why the trial ended or was stopped (early)

Does your paper address CONSORT subitem 14b? *

Copy and paste relevant sections from the manuscript (include quotes in quotation marks "like this" to indicate direct quotes from your manuscript), or elaborate on this item by providing additional information not in the ms, or briefly explain why the item is not applicable/relevant for your study

This item does not apply to the present study

15) A table showing baseline demographic and clinical characteristics for each group

NPT: When applicable, a description of care providers (case volume, qualification, expertise, etc.) and centers (volume) in each group

Does your paper address CONSORT subitem 15? *

Copy and paste relevant sections from the manuscript (include quotes in quotation marks "like this" to indicate direct quotes from your manuscript), or elaborate on this item by providing additional information not in the ms, or briefly explain why the item is not applicable/relevant for your study

". Table 1 presents the demographics of the 36 participants who completed the study and table 2 presents the participants' baseline characteristics according to group allocation."

15-i) Report demographics associated with digital divide issues

In ehealth trials it is particularly important to report demographics associated with digital divide issues, such as age, education, gender, social-economic status, computer/Internet/ehealth literacy of the participants, if known.

	1	2	3	4	5	
subitem not at all important	(selected)					essential

Clear selection

Does your paper address subitem 15-i? *

Copy and paste relevant sections from the manuscript (include quotes in quotation marks "like this" to indicate direct quotes from your manuscript), or elaborate on this item by providing additional information not in the ms, or briefly explain why the item is not applicable/relevant for your study

Not applicable as this is not a mobile APP intervention

16) For each group, number of participants (denominator) included in each analysis and whether the analysis was by original assigned groups

16-i) Report multiple “denominators” and provide definitions

Report multiple “denominators” and provide definitions: Report N’s (and effect sizes) “across a range of study participation [and use] thresholds” [1], e.g., N exposed, N consented, N used more than x times, N used more than y weeks, N participants “used” the intervention/comparator at specific pre-defined time points of interest (in absolute and relative numbers per group). Always clearly define “use” of the intervention.

	1	2	3	4	5	
subitem not at all important					(selected)	essential

Clear selection

Does your paper address subitem 16-i? *

Copy and paste relevant sections from the manuscript (include quotes in quotation marks "like this" to indicate direct quotes from your manuscript), or elaborate on this item by providing additional information not in the ms, or briefly explain why the item is not applicable/relevant for your study

"From October 1, 2020 to August 31, 2022, 7,160 patients received hand therapy rehabilitation at the two hospitals, from which 110 were eligible to enter the trial and 38 consented to participate (participation rate of 35%). The trial retention rate was 95% (36/38). The retention rate for the control group was 100% and for the intervention group it was 89% (17/19). Two participants declined to participate after the first assessment due to disagreement with measures in place to control the spread of the pandemic. "

16-ii) Primary analysis should be intent-to-treat

Primary analysis should be intent-to-treat, secondary analyses could include comparing only "users", with the appropriate caveats that this is no longer a randomized sample (see 18-i).

	1	2	3	4	5	
subitem not at all important	○	○	○	○	◉	essential

Clear selection

Does your paper address subitem 16-ii?

Copy and paste relevant sections from the manuscript (include quotes in quotation marks "like this" to indicate direct quotes from your manuscript), or elaborate on this item by providing additional information not in the ms, or briefly explain why the item is not applicable/relevant for your study

"The analysis was conducted using an intention-to-treat principle. "

17a) For each primary and secondary outcome, results for each group, and the estimated effect size and its precision (such as 95% confidence interval)

Does your paper address CONSORT subitem 17a? *

Copy and paste relevant sections from the manuscript (include quotes in quotation marks "like this" to indicate direct quotes from your manuscript), or elaborate on this item by providing additional information not in the ms, or briefly explain why the item is not applicable/relevant for your study

"Table 4 displays the change-from-baseline outcome variables for both groups."
"Table 4 displays the change-from-baseline outcome variables for both groups. Comparing the change-from-baseline between groups revealed that in 63% (12/19) of the outcome variables, the change was greater in the intervention group that used the FEPSim® than in the control group; however, statistically significant differences between groups were observed in only two of the outcome variables 11% (2/19), specifically grip strength and passive wrist flexion, both in favor of the intervention group. The MCID was 0.96 for grip strength and 1.08 for passive wrist flexion. For the PRWE scale, including the pain and function subscales, as well as the overall summative scale, the change-from-baseline was not in the desired direction (unfavorable for the FEPSim®). However, no statistically significant difference was observed for these outcome variables."

17a-i) Presentation of process outcomes such as metrics of use and intensity of use

In addition to primary/secondary (clinical) outcomes, the presentation of process outcomes such as metrics of use and intensity of use (dose, exposure) and their operational definitions is critical. This does not only refer to metrics of attrition (13-b) (often a binary variable), but also to more continuous exposure metrics such as "average session length". These must be accompanied by a technical description how a metric like a "session" is defined (e.g., timeout after idle time) [1] (report under item 6a).

	1	2	3	4	5	
subitem not at all important	○	○	○	○	●	essential

Clear selection

Does your paper address subitem 17a-i?

Copy and paste relevant sections from the manuscript (include quotes in quotation marks "like this" to indicate direct quotes from your manuscript), or elaborate on this item by providing additional information not in the ms, or briefly explain why the item is not applicable/relevant for your study

"Mean number of treatment sessions received per group are highlighted in table 3. We found no statistically significant differences in the number of weeks, the number of sessions, nor the number of days between the pre-test and post-test between groups. All the participants in the intervention group received the treatment with the FEPSim®. On average, the therapists used the FEPSim® in 89% of the sessions in the intervention group."

17b) For binary outcomes, presentation of both absolute and relative effect sizes is recommended

Does your paper address CONSORT subitem 17b? *

Copy and paste relevant sections from the manuscript (include quotes in quotation marks "like this" to indicate direct quotes from your manuscript), or elaborate on this item by providing additional information not in the ms, or briefly explain why the item is not applicable/relevant for your study

"The overall fixed-effects model calculated a small effect size. That is, we obtained a statistically significant test for Cohen's d = 0.189 (Z = 2.450, p = .014, 95% CI [0.038, 0.340]) with a standard error of 0.077. "

18) Results of any other analyses performed, including subgroup analyses and adjusted analyses, distinguishing pre-specified from exploratory

Does your paper address CONSORT subitem 18? *

Copy and paste relevant sections from the manuscript (include quotes in quotation marks "like this" to indicate direct quotes from your manuscript), or elaborate on this item by providing additional information not in the ms, or briefly explain why the item is not applicable/relevant for your study

"The MCID was 0.96 for grip strength and 1.08 for passive wrist flexion. "

18-i) Subgroup analysis of comparing only users

A subgroup analysis of comparing only users is not uncommon in ehealth trials, but if done, it must be stressed that this is a self-selected sample and no longer an unbiased sample from a randomized trial (see 16-iii).

	1	2	3	4	5	
subitem not at all important	(selected)	○	○	○	○	essential

Clear selection

Does your paper address subitem 18-i?

Copy and paste relevant sections from the manuscript (include quotes in quotation marks "like this" to indicate direct quotes from your manuscript), or elaborate on this item by providing additional information not in the ms, or briefly explain why the item is not applicable/relevant for your study

Not applicable as this is not a mobile APP intervention

19) All important harms or unintended effects in each group

(for specific guidance see CONSORT for harms)

Does your paper address CONSORT subitem 19? *

Copy and paste relevant sections from the manuscript (include quotes in quotation marks "like this" to indicate direct quotes from your manuscript), or elaborate on this item by providing additional information not in the ms, or briefly explain why the item is not applicable/relevant for your study

No harms were identified in this trial

19-i) Include privacy breaches, technical problems

Include privacy breaches, technical problems. This does not only include physical "harm" to participants, but also incidents such as perceived or real privacy breaches [1], technical problems, and other unexpected/unintended incidents. "Unintended effects" also includes unintended positive effects [2].

	1	2	3	4	5	
subitem not at all important	(●)	○	○	○	○	essential

Clear selection

Does your paper address subitem 19-i?

Copy and paste relevant sections from the manuscript (include quotes in quotation marks "like this" to indicate direct quotes from your manuscript), or elaborate on this item by providing additional information not in the ms, or briefly explain why the item is not applicable/relevant for your study

Not applicable as this is not a mobile APP intervention

19-ii) Include qualitative feedback from participants or observations from staff/researchers

Include qualitative feedback from participants or observations from staff/researchers, if available, on strengths and shortcomings of the application, especially if they point to unintended/unexpected effects or uses. This includes (if available) reasons for why people did or did not use the application as intended by the developers.

	1	2	3	4	5	
subitem not at all important	○	○	○	○	◉	essential

Clear selection

Does your paper address subitem 19-ii?

Copy and paste relevant sections from the manuscript (include quotes in quotation marks "like this" to indicate direct quotes from your manuscript), or elaborate on this item by providing additional information not in the ms, or briefly explain why the item is not applicable/relevant for your study

"The therapists' acceptance of this device is also backed up by the qualitative results of another study published elsewhere.[14]"

DISCUSSION

22) Interpretation consistent with results, balancing benefits and harms, and considering other relevant evidence

NPT: In addition, take into account the choice of the comparator, lack of or partial blinding, and unequal expertise of care providers or centers in each group

22-i) Restate study questions and summarize the answers suggested by the data, starting with primary outcomes and process outcomes (use)

Restate study questions and summarize the answers suggested by the data, starting with primary outcomes and process outcomes (use).

	1	2	3	4	5	
subitem not at all important	○	○	○	○	◉	essential

Clear selection

Does your paper address subitem 22-i? *

Copy and paste relevant sections from the manuscript (include quotes in quotation marks "like this" to indicate direct quotes from your manuscript), or elaborate on this item by providing additional information not in the ms, or briefly explain why the item is not applicable/relevant for your study

"Trial feasibility
Overall, the research design is feasible for a definitive RCT and offers valuable information for ongoing and future studies. The high trial retention and compliance showed that both the therapists and participants from the two hand therapy services agreed to incorporate the FEPSim® into their hand therapy treatments. The therapists' acceptance of this device is also backed up by the qualitative results of another study published elsewhere.[14]
Clinical effectiveness of adding the FEPSim® device to hand therapy
This study provides evidence of the potential clinical benefits of incorporating the FEPSim® device into hand therapy. Both groups improved with the hand therapy treatment in around eight weeks. However, the FEPSim® group showed improvement in more outcome variables, associated with a statistically significantly higher effect size. For grip strength and passive wrist flexion, the change-from-baseline was statistically significant in favor of the FEPSim®. In the intervention group, 76.4% of participants showed a change equal to or greater than the MCID for grip strength, and 88.2% for passive wrist flexion. Grip strength is a commonly used objective proxy of hand function due to its strong correlation with overall hand performance and functionality [15,16]. Grip strength is a composite measure that reflects the integrated function of various hand muscles and joints, capturing both intrinsic and extrinsic muscle function, is clinically relevant and is associated with various health outcomes.[17] Furthermore, sufficient strength in the upper limb is related to the ability to adequately perform many activities of daily living.
"

22-ii) Highlight unanswered new questions, suggest future research

Highlight unanswered new questions, suggest future research.

	1	2	3	4	5	
subitem not at all important	○	○	○	○	◉	essential

Clear selection

Does your paper address subitem 22-ii?

Copy and paste relevant sections from the manuscript (include quotes in quotation marks "like this" to indicate direct quotes from your manuscript), or elaborate on this item by providing additional information not in the ms, or briefly explain why the item is not applicable/relevant for your study

"Recommendations for a definitive RCT
Based on the results of this feasibility trial, the authors make the following recommendations for a definitive RCT. First, to investigate a larger sample of diagnoses, including the emerging eight diagnoses, from the outset to promote sample size and a more robust evaluation. Second, we recommend having three measurement points pre-test (Week 0), post-test (immediately after discharge) and follow-up at six months. This recommendation is based on the observation that the percentage of participants who were discharged from hand therapy services increased during the trial, and that participants may have been discharged at different points during the proposed protocol. Third, considering that the sample size calculation showed that a total of 350 participants will be required and that we had a drop-out rate of 10%, 385 participants will be required to participate in a definitive RCT. Considering that the participation rate of this feasibility trial was 34.5%, for a definitive trial the required accessible population will be 1,116 patients. To ensure an adequate sample size for a definitive RCT that recruits participants over a two-year period, it is recommended that recruitment is from ten settings. This is based on the observation that the feasibility trial admitted approximately five patients per month who potentially met the inclusion criteria, resulting in an estimated 47 eligible patients per month (1,116 patients/24 months). It is important to note that these conclusions were reached under challenging circumstances, as the data used to inform this recommendation was collected in hospital settings during the COVID-19 pandemic."

20) Trial limitations, addressing sources of potential bias, imprecision, and, if relevant, multiplicity of analyses

20-i) Typical limitations in ehealth trials

Typical limitations in ehealth trials: Participants in ehealth trials are rarely blinded. Ehealth trials often look at a multiplicity of outcomes, increasing risk for a Type I error. Discuss biases due to non-use of the intervention/usability issues, biases through informed consent procedures, unexpected events.

	1	2	3	4	5	
subitem not at all important	○	○	○	○	●	essential

Clear selection

Does your paper address subitem 20-i? *

Copy and paste relevant sections from the manuscript (include quotes in quotation marks "like this" to indicate direct quotes from your manuscript), or elaborate on this item by providing additional information not in the ms, or briefly explain why the item is not applicable/relevant for your study

"Study Limitations
It is worth noting that the study has some limitations. Given the limited sample size and low statistical power, caution should be exercised when interpreting the results; it is important to consider these limitations in the context of its feasibility nature and the power calculation conducted for a future definitive trial. Another important consideration is that the two variables showing statistically significant differences between groups—grip strength and passive wrist flexion—had missing values in 19% and 14% of participants, respectively. These missing pretest values were imputed using the single imputation method of carrying the last observation backward. While this approach is widely used, it, like all imputation methods, has inherent limitations. The COVID-19 pandemic had a significant impact on the trial, with different hospital policies for COVID-19 prevention and control, resulting in varying recruitment launch dates and the closure of one hospital's hand therapy service. This led to a significant reduction in the number of potential participants who met the inclusion criteria. In addition, the data collection was affected, as the trial was conducted over several months during the pandemic. A study by Ivy et al. investigated the pandemic's impact on hand therapy services; they reported a decrease in caseload for 98% of the 719 surveyed hand therapists, with 46% providing telehealth services.[22] However, it is important to note that the impact of the pandemic on the results of the study may differ in a post-pandemic world, where hand therapy services may have returned to pre-pandemic levels. Lastly, as no follow-up measurement points were incorporated in the study, the long-term effects cannot be determined; thus, the results are limited to their generalizability to the short-term."

21) Generalisability (external validity, applicability) of the trial findings

NPT: External validity of the trial findings according to the intervention, comparators, patients, and care providers or centers involved in the trial

21-i) Generalizability to other populations

Generalizability to other populations: In particular, discuss generalizability to a general Internet population, outside of a RCT setting, and general patient population, including applicability of the study results for other organizations

	1	2	3	4	5	
subitem not at all important	○	○	○	○	◉	essential

Clear selection

Does your paper address subitem 21-i?

Copy and paste relevant sections from the manuscript (include quotes in quotation marks "like this" to indicate direct quotes from your manuscript), or elaborate on this item by providing additional information not in the ms, or briefly explain why the item is not applicable/relevant for your study

"To ensure an adequate sample size for a definitive RCT that recruits participants over a two-year period, it is recommended that recruitment is from ten settings. "

21-ii) Discuss if there were elements in the RCT that would be different in a routine application setting

Discuss if there were elements in the RCT that would be different in a routine application setting (e.g., prompts/reminders, more human involvement, training sessions or other co-interventions) and what impact the omission of these elements could have on use, adoption, or outcomes if the intervention is applied outside of a RCT setting.

	1	2	3	4	5	
subitem not at all important	○	○	○	○	◉	essential

Clear selection

Does your paper address subitem 21-ii?

Copy and paste relevant sections from the manuscript (include quotes in quotation marks "like this" to indicate direct quotes from your manuscript), or elaborate on this item by providing additional information not in the ms, or briefly explain why the item is not applicable/relevant for your study

"It is important to note that these conclusions were reached under challenging circumstances, as the data used to inform this recommendation was collected in hospital settings during the COVID-19 pandemic."

OTHER INFORMATION

23) Registration number and name of trial registry

Does your paper address CONSORT subitem 23? *

Copy and paste relevant sections from the manuscript (include quotes in quotation marks "like this" to indicate direct quotes from your manuscript), or elaborate on this item by providing additional information not in the ms, or briefly explain why the item is not applicable/relevant for your study

Trail registration: Number Registry ISRCTN13656014;
https://www.isrctn.com/ISRCTN13656014

24) Where the full trial protocol can be accessed, if available

Does your paper address CONSORT subitem 24? *

Cite a Multimedia Appendix, other reference, or copy and paste relevant sections from the manuscript (include quotes in quotation marks "like this" to indicate direct quotes from your manuscript), or elaborate on this item by providing additional information not in the ms, or briefly explain why the item is not applicable/relevant for your study

"In order to investigate the areas of uncertainty regarding a future definitive randomized controlled trial (RCT), this feasibility study, of which the protocol has been published previously,[5] " (5. Miguel-Cruz A, Guptill C, Gregson G, Ladurner AM, Holmes C, Yeung D, Siebert J, Dziwenko G, Rincón AR. Determining the effectiveness of a new device for hand therapy (The FEPSim Device): Feasibility protocol for a randomized controlled trial study. JMIR Res Protoc JMIR Publications Inc.; 2021 May 27;10(5):e22145. PMID:34042597)

25) Sources of funding and other support (such as supply of drugs), role of funders

Does your paper address CONSORT subitem 25? *

Copy and paste relevant sections from the manuscript (include quotes in quotation marks "like this" to indicate direct quotes from your manuscript), or elaborate on this item by providing additional information not in the ms, or briefly explain why the item is not applicable/relevant for your study

"This work was supported by Alberta Innovates in 2018/2019 AICE Accelerating Innovation Into Care [grant number G2019000520]."

X27) Conflicts of Interest (not a CONSORT item)

X27-i) State the relation of the study team towards the system being evaluated

In addition to the usual declaration of interests (financial or otherwise), also state the relation of the study team towards the system being evaluated, i.e., state if the authors/evaluators are distinct from or identical with the developers/sponsors of the intervention.

	1	2	3	4	5	
subitem not at all important	○	○	○	○	◉	essential

Clear selection

Does your paper address subitem X27-i?

Copy and paste relevant sections from the manuscript (include quotes in quotation marks "like this" to indicate direct quotes from your manuscript), or elaborate on this item by providing additional information not in the ms, or briefly explain why the item is not applicable/relevant for your study

"None declared."

Your answer must have a minimum of 25 characters.

About the CONSORT EHEALTH checklist

As a result of using this checklist, did you make changes in your manuscript? *

- ○ yes, major changes
- ○ yes, minor changes
- ◉ no

What were the most important changes you made as a result of using this checklist?

No changes were made to the manuscript

How much time did you spend on going through the checklist INCLUDING making changes in your manuscript *

The form took approximately 2 hours to complete. I found the process to be quite time-consuming, as the form is not very user-friendly. Filling it out required a significant amount of effort, primarily involving copying and pasting information directly from my manuscript.

I would suggest that the group and journal review the relevance and utility of this form to ensure it adds value to the submission process. Streamlining the form could save authors considerable time and make the process more efficient.

Thank you for considering this feedback.

As a result of using this checklist, do you think your manuscript has improved? *

- ○ yes
- ◉ no
- ○ Other:

Would you like to become involved in the CONSORT EHEALTH group?

This would involve for example becoming involved in participating in a workshop and writing an "Explanation and Elaboration" document

- ○ yes
- ◉ no
- ○ Other:

Clear selection

Any other comments or questions on CONSORT EHEALTH

Your answer

STOP - Save this form as PDF before you click submit

To generate a record that you filled in this form, we recommend to generate a PDF of this page (on a Mac, simply select "print" and then select "print as PDF") before you submit it.

When you submit your (revised) paper to JMIR, please upload the PDF as supplementary file.

Don't worry if some text in the textboxes is cut off, as we still have the complete information in our database. Thank you!

Final step: Click submit !

Click submit so we have your answers in our database!

Submit Clear form

Never submit passwords through Google Forms.

This form was created outside of your domain. - Terms of Service - Privacy Policy

Does this form look suspicious? Report

Google Forms
